# The Application of Nano Drug Delivery Systems in Female Upper Genital Tract Disorders

**DOI:** 10.3390/pharmaceutics16111475

**Published:** 2024-11-19

**Authors:** Daniélle van Staden, Minja Gerber, Hendrik J. R. Lemmer

**Affiliations:** Centre of Excellence for Pharmaceutical Sciences (PharmacenTM), North-West University, Potchefstroom 2531, South Africa; dvanstaden711@gmail.com (D.v.S.); minja.gerber@nwu.ac.za (M.G.)

**Keywords:** drug delivery, endometriosis, exosomes, genitourinary cancer, infertility, genital tract disorders, nanoparticle, nanotechnology, polycystic ovary syndrome, reproductive health

## Abstract

The prevalence of female reproductive system disorders is increasing, especially among women of reproductive age, significantly impacting their quality of life and overall health. Managing these diseases effectively is challenging due to the complex nature of the female reproductive system, characterized by dynamic physiological environments and intricate anatomical structures. Innovative drug delivery approaches are necessary to facilitate the precise regulation and manipulation of biological tissues. Nanotechnology is increasingly considered to manage reproductive system disorders, for example, nanomaterial imaging allows for early detection and enhances diagnostic precision to determine disease severity and progression. Additionally, nano drug delivery systems are gaining attention for their ability to target the reproductive system successfully, thereby increasing therapeutic efficacy and decreasing side effects. This comprehensive review outlines the anatomy of the female upper genital tract by highlighting the complex mucosal barriers and their impact on systemic and local drug delivery. Advances in nano drug delivery are described for their sustainable therapeutic action and increased biocompatibility to highlight the potential of nano drug delivery strategies in managing female upper genital tract disorders.

## 1. Introduction

The female reproductive system can be anatomically divided into two main regions. The lower region includes the vaginal canal and ectocervix [1]. The upper region consists of the endocervix, uterus, fallopian tubes, and ovaries [1]. The epithelium lining the vaginal canal is structured in a stratified squamous arrangement, continuing to the ectocervix, and creating a barrier harboring multiple epithelial cell layers to guard against external pathogenic organisms [2,3]. The stratified squamous epithelium originates at a basement membrane comprising progenitor cells, that ages into a fully developed keratinized epithelium [2,3]. This arrangement facilitates the formation of multiple tight junction levels that prevent pathogen infiltration while permitting the closest layer to the lumen to remain permeable to transudate from the blood and shed mucosal cells [4]. The literature describes a “transformation zone”, marking the transition from the ectocervix to the endocervix, where the epithelial structure transitions from a stratified squamous to a simple columnar epithelial structure [4]. The columnar epithelial layer proceeds into the endometrium, thus rendering the female upper genital tract (UGT) more susceptible to pathogens [4]. This susceptibility is especially pertinent in cases of cervical ectopy, commonly found in young, pregnant, or oral-contraceptive-using women, where the endocervix protrudes into the vaginal canal [2,4]. This protrusion heightens the vulnerability of the female UGT to infection and has been linked to the acquisition of sexually transmitted infections such as human immunodeficiency virus (HIV) and human papillomavirus [5]. In addition to the physical epithelial barrier, cervicovaginal mucus, originating from goblet cells of the endocervix, combines with cellular debris, vaginal transudate, and immune components to form a matrix that entraps potential pathogens, averting their interaction with the epithelial layer and potential target cells [6]. Immune components, including secreted proteins such as immunoglobulins, antimicrobial peptides, and proteases, are integral to this process, binding to pathogens and facilitating their clearance from the female genital tract via mucus flow [4,7].

Apart from infections, the UGT can be impacted by divergent health problems such as inflammation, tumors and adhesions [8,9]. In terms of drug delivery, the multifaceted nature of the UGT adds to the complexity of achieving targeted and efficient drug delivery [9]. The uterus is a dynamic physiological environment influenced by inter-individual immune composition, hormones and the microbiome which changes during the intrinsic ageing process [4,9,10]. Moreover, UGT diseases can originate from surrounding tissues [9]. Hence, the treatment of female UGT disorders ideally requires targeted drug delivery to improve efficacy and decrease the incidence of adverse reactions [9,11]. Moreover, the advantages of targeted drug delivery can address concerns involving pregnant patients with UGT disorders to aid in sustaining pregnancy with a reduced risk of congenital malformation [12]. Female UGT diseases annually impact the lives of large numbers of women [13]. Consequently, female genital tract disorders add to high economic burdens and decrease the quality of life due to serious health repercussions [13]. Epidemiological data reveal a recent increase in the prevalence of female reproductive system diseases, particularly among younger age cohorts [11,14]. These conditions have the potential to cause organ damage and functional impairments, and can adversely affect reproductive capability, potentially leading to infertility [11,14]. For instance, pelvic inflammatory disease (PID), a significant cause of gynecologic hospitalization, encompasses a spectrum of conditions like endometritis, parametritis, chorioamnionitis, salpingitis, oophoritis, peritonitis, tubo-ovarian and pelvic abscess, and septicemia [9].

PID is estimated to affect approximately 2.5 million women in the United States, ranging in age from 18 to 44 years [9]. A study investigating the incidence of PID and ectopic pregnancy (EP) suggested that the increased prevalent cases of active PID may be attributed to overall global population growth. At the same time, reducing the age-standardized prevalence rate (ASPR) of PID indicates advancements in the medical prevention, management, and treatment of PID-related conditions [15]. These declining values correlate with public health endeavors aimed at controlling the prevalence of sexually transmitted infections, specifically *Chlamydia trachomatis* and *Neisseria gonorrhoeae* infections, which exhibited a declining trend from 1990 to 2019 [15]. The decline in cases of EP may be associated with the decreasing rate of PID. This correlation may stem from the selective loss of ciliated epithelial cells along the fallopian tube epithelium due to infection, which could impede ovum transport and lead to EP [15,16]. In 2019, the Sub-Saharan Africa region exhibited the highest ASPRs of PID, largely attributed to insufficient healthcare and sexual health education [15,17]. However, there has been a notable decrease in PID rates in these regions since 1990, indicative of progress in PID and sexually transmitted infection detection, diagnosis, and treatment [15]. In 2019, Niger, Burkina Faso, and Gambia had the highest ASPRs of PID in Sub-Saharan Africa, while Mali and Cameroon, in the same region, demonstrated the most significant decreases in PID rates [15]. The reduced incidence of PID in Western Europe can be attributed to its advanced medical care system [15]. However, despite the declining trajectory of PID and EP, the literature still reports that clinicians may experience limited therapeutic options available to treat female reproductive disorders effectively [18]. Therefore, the literature signifies a clear need for the development of drug delivery systems capable of targeting female UGT to improve health outcomes while decreasing side effects.

Recent advancements adding to the understanding of UGT physiology and pathology, combined with biomedical innovations, have prompted the development of targeted drug delivery technologies for addressing female health concerns [9]. Conventional treatment of female reproductive system diseases is hindered by suboptimal drug efficacy, which can add to significant side-effect profiles [11,14]. Thus, the imperative pursuit of more potent and precise treatment strategies is reported in the literature [11]. Nanotechnology presents a promising frontier to aid in female reproductive disorders, by facilitating targeted drug delivery via carriers with an inherent capacity to improve positive treatment outcomes while reducing adverse effects [19,20]. Additionally, nanoparticles are considered promising molecular markers in diagnostics due to their enhanced precision in disease detection, consequently facilitating a prompt diagnosis and reliable estimations of progress of the established lesions [21]. Nano-based diagnostic technology utilizes materials in the nanometer-scale range (0.1–100.0 nm) by incorporating the principles of classical and quantum mechanics, along with modern technologies such as micro-electronics, scanning tunnelling microscopy, and nuclear analysis techniques [11,22]. The principal branches of nanotechnology encompass nano-system physics, nano-scale chemistry, nanomaterials science, nano-biology, nano-electronics, nano-fabrication, and nano-mechanics [11]. Leveraging nanotechnology offers the opportunity to devise more exact and efficient treatment modalities, including targeted drug delivery and tissue engineering [23].

In this review, we provide an overview of the anatomy, physiology, and mucosal barriers of the female UGT. This overview is followed by a discussion on the different approaches of systemic and local drug delivery involving the treatment of female reproductive disorders, and then expands on advances regarding nanoparticles to address the shortcomings of conventional drug delivery systems when targeting the female reproductive system. In conclusion, the safety implications of employing nano-sized drug delivery systems and future prospects are presented.

## 2. Anatomy, Physiology, and Mucosal Barriers of the Female Upper Genital Tract

**Figure 1:**
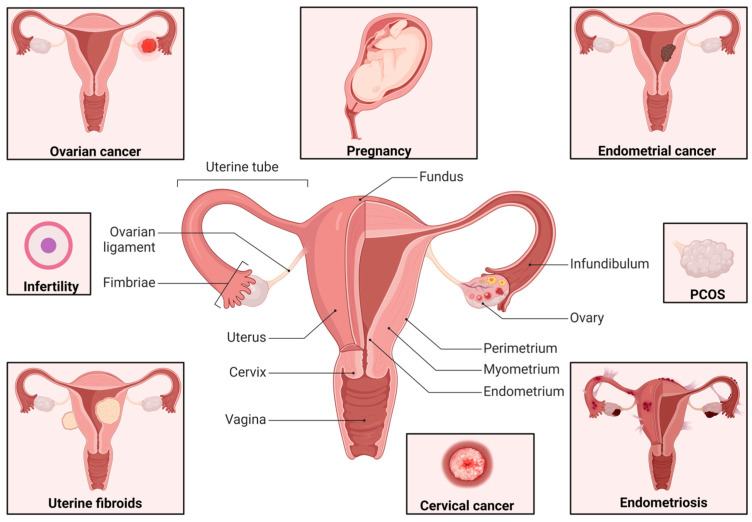
Anatomy of the female upper genital tract with inserts illustrating conditions affecting the female upper genital tract (PCOS: polycystic ovary syndrome). Created in BioRender. Van staden, D. (2024) BioRender.com/m21v373 [11].

Optimized targeting of the female UGT requires a comprehensive understanding of the unique anatomy, physiology, and pathophysiology of different tissues forming part of the female reproductive tract (refer to Figure 1) [9]. Particularly crucial is an understanding of the unique mucosal barriers within the UGT, which undergo significant changes throughout the menstrual cycle, menopause, and pregnancy, unlike mucosal sites in other bodily systems such as the respiratory and gastrointestinal tracts [24,25,26]. When considering the anatomy of the UGT, the cervix is considered the anatomical link between the lower and upper female genital tract. As an anatomic site, the cervix is divided into three compartments, known as the ectocervix, the cervical transformation zone, and the endocervix [27]. The cervix is an area with known pathologies such as cervical cancer, as presented in Figure 1 [27,28,29].

As seen in Figure 1, the endocervix connects the lower genital tract and the uterus. The literature describes the uterus as the largest structure and primary reproductive fibromuscular organ of the female UGT involved in essential female reproductive functions [30]. The uterus comprises the serosa, myometrium, and endometrium (uterine mucosa). The uterine mucosa produces uterine fluid subjected to cyclical changes hormonally regulated during the menstrual cycle and pregnancy [9,31]. The menstrual cycle involves three principal phases that impact endometrial thickness, namely menstrual, proliferative, and secretory phases [31]. These phases are intricately involved in hormonal regulation, primarily governed by estrogen and progesterone, influencing the endometrium [32]. The ovarian artery, uterine artery, and uterine vein facilitate vascularization of the uterus [30,33]. A myriad of disorders is linked to the uterus [34,35,36,37,38,39]. As an example, endometriosis presents chronic inflammation at affected sites that can involve endometrial tissue development in regions like the fallopian tubes, rectum, vagina, ovaries, and pelvic peritoneum [40]. Adenomyosis entails the growth of endometrial cells within the uterine walls, resulting in a thickened endometrium [41]. Uterine fibroids are non-cancerous growths dependent on estrogen and progesterone, originating from the myometrium [42]. Adhesions consisting of fibrous, scar-like tissues can transpire naturally or arise after surgical interventions, causing fastening to tissues and organs [43].

Uterine tubes and ovaries are contiguous to the uterus [9]. Uterine tubes, also known as the oviducts or fallopian tubes, serve as a crucial anatomical conduit between the ovary and the uterus [44]. The fallopian tubes, measuring approximately 11.0 cm in length, are fluid-filled sacs situated near the ovaries. The fimbriae, positioned at the distal end of the fallopian tubes, guides the released ovum from the ovaries [9]. The structure of the fallopian tubes comprises a mucosal layer, followed by a muscular layer, and a serosa layer [45]. These anatomical components serve as potential sites for conditions such as cancer, infections, inflammation, EP, and tubal blockage, all of which can culminate in infertility [45]. Congenital or acquired irregularities in these structures can directly impede the efficient transportation of ova or spermatozoa, depending on the specific anatomical locus affected [44]. Uterine tube pathology is increasingly diagnosed and likely underpins numerous instances of infertility typified by repeated medical visits without discovering discernible manifestations or palpable irregularities within the reproductive tract [46]. The fallopian tubes link the ovaries to the uterus. The ovaries are a paired set of organs situated on the lateral aspects of the pelvis, possessing distinct layers such as the outer layer, capsule, cortex, and medulla [9]. These organs are susceptible to various conditions, including infections, ovarian cancer, premature ovarian failure, and polycystic ovary syndrome (PCOS). Premature ovarian failure is typified by ovarian dysfunction and a diminished ovarian reserve, stemming from pathological factors or natural aging [9,47]. Conversely, PCOS is a multifaceted disorder characterized by hyperandrogenism and the presence of polycystic ovaries, often contributing to fertility challenges [48].

The female UGT is lined with a mucosal barrier to prevent chemical, microbial, and mechanical injury [9]. Notable examples include tight junctions, adherents, and desmosomes expressed by uterine epithelial cells that protect the underlying mucosa [49,50]. This mucosal lining also synthesizes various antimicrobial peptides under hormonal regulation to combat invading pathogens originating from the lower genital tract or systemic circulation [49,50]. Moreover, the mucosal lining accommodates several immune cells, including natural killer cells, macrophages, CD8+ T cells, and B cells, serving a dual purpose by aiding in protection and reproduction [51]. Consequently, it is imperative to consider the disruption of local immune function inflicted by drug delivery systems [9]. Research reveals that the female UGT harbors a diverse microbiome [51]. In this microbiome, *Lactobacillus* is identified as the prominent genus; therefore, studies have found that a non-*Lactobacillus*-dominated microbiota negatively impacts female reproductive health [52,53]. Disruptions in the uterine microbiome homeostasis may lead to complications in conception, infertility, preterm birth (PTB), and endometritis [10,52,53,54,55]. Hence, any dysbiosis resulting from external stimuli, such as drug delivery systems, necessitates careful consideration [9]. However, drug delivery in the female UGT is complex, as one must find a balance between avoiding dysbiosis while designing vehicles capable of crossing the protective mucosal barrier lining the female UGT.

## 3. Mucosal Barrier Lining the Female Upper Genital Tract

**Figure 2:**
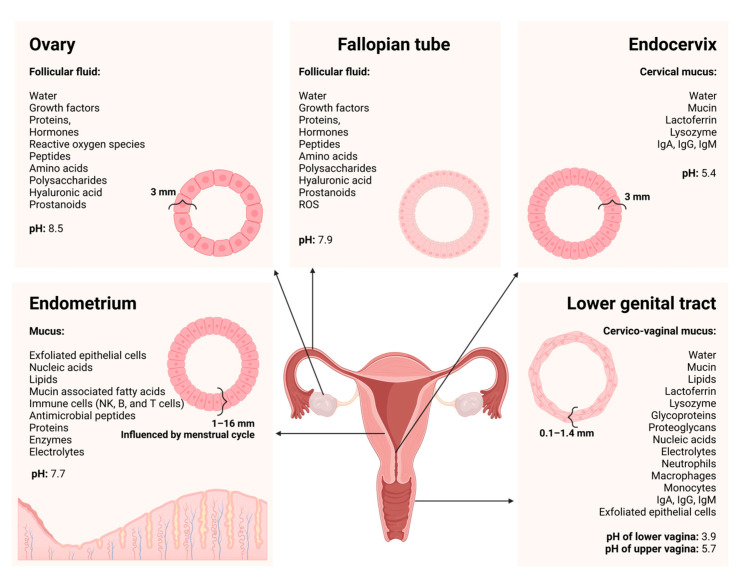
Physiological properties of the female reproductive tract that can impact drug delivery (IgA: immunoglobulin A, IgG: immunoglobulin G, IgM: immunoglobulin M, NK: natural killer, ROS: reactive oxygen species). Created in BioRender. Van staden, D. (2024), https://BioRender.com/g25g365 [6,9,63,64,65,66,67].

The mucosal barrier lining the female UGT presents a steric hindrance to pathogens and drug entities by protectively trapping unwarranted substances within the mucus network [6]. Drug carriers can be tailored to allow for swift diffusion across mucus networks to avoid trapping the drug within the mucus, followed by drug degradation before reaching the absorptive epithelial membrane site [56]. Hence, mucus permeation is a major requirement for efficacious drug targeting in the female UGT [9]. Several strategies have been proposed and validated in the literature to facilitate the crossing of mucosal barriers [56]. Examples include nanoparticulate systems, incorporating enzymatically active components or systems able to provide a “slippery surface” and zeta-potential-changing drug delivery carriers [57,58,59,60]. Therefore, to design drug delivery systems that target the UGT effectively, mucosal barriers within the UGT should be considered as these mucosal barriers distinctly differ from mucosal linings of the respiratory and gastrointestinal tracts [9]. Apart from taking into consideration the protective mucosal barrier, the endometrium changes throughout the menstrual cycle, during menopause, and in pregnancy [61,62]. To provide a foundation for understanding the complex physiological properties of the female genital tract, Figure 2 summarizes the divergent fluid and mucus composition throughout the female genital tract, illustrating different cell types associated with individual genital tract regions and indicating alteration in regional pH values.

**Figure 3:**
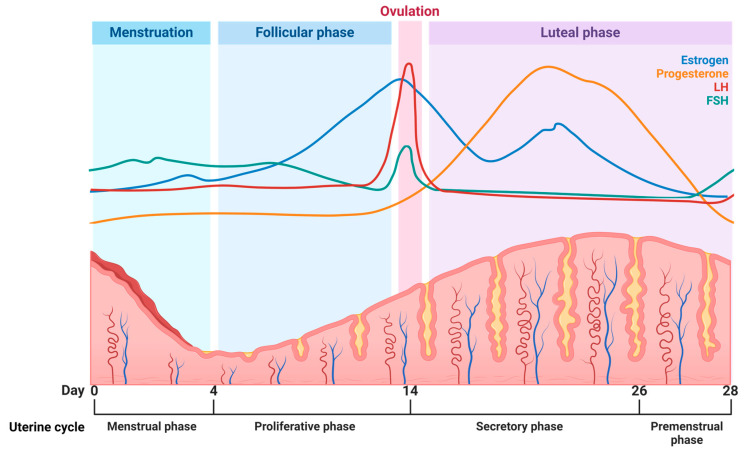
Hormonal changes impacting endometrial evolvement during the menstrual cycle (FSH: follicle-stimulating hormone, LH: luteinizing hormone). Created in BioRender. Van staden, D. (2024) BioRender.com/s70l858 [69].

Apart from the regional changes (as displayed in Figure 2), drug delivery in the female UGT can be hindered by the local epithelium, metabolizing enzymes, diverse tissue composition, microbiota, drug transporters, hormonal fluctuation, blood perfusion, lymphatic drainage, and complex composition of dynamic fluids [9,68]. Importantly, the lower genital tract has significantly different physiological properties compared to the UGT, implicating that expertise is required to facilitate localized drug delivery to the UGT via vaginal administration. Additionally, sterility requirements, anatomic accessibility and non-target drug exposure add to the complexity of effectively targeting female UGT disorders [9]. Practically, patient compliance is constrained by personal preference, social acceptability, and therapy cost, which add to the high burden of the insufficient treatment of UGT disorders [9]. Factors that impact drug pharmacokinetics in the female UGT include tissue permeability, drug transporters, protein binding, enzymatic activity, and fluid retention within the UGT [9]. Importantly, these factors are influenced by the stage of the menstrual cycle and pregnancy, and can render variable drug efficacy profiles [9,24,26,31,55]. This is evident from the varying thickness of uterine tissues due to progesterone and/or progestin exposure, the presence of inflammation and infection, as well as normal physiological changes occurring during the menstrual cycle [9,24,31]. During a normal menstrual cycle, the stratum functionalis of the endometrium is discarded during each cycle, followed by rapid regeneration involving an inflammatory resolution and tissue remodeling, as illustrated in Figure 3 [24,62]. Interestingly, the physiological changes observed during the menstrual cycle can potentially impact drug pharmacokinetics [69]. Therefore, Table 1 and Figure 3 provide a mutual representation of the physiological changes and the impact thereof on drug delivery.

As illustrated by Table 1 and Figure 3, the hormonal changes that occur during the menstrual cycle affect multiple body systems, but medical treatment, including medication dosing, is mostly standardized for both men and women [70]. It is important to emphasize that we have a limited understanding of how drugs affect women differently based on their unique hormone profiles. Even though the natural hormonal variations during menstruation affect women’s physiology and brain chemistry, medication dosages do not take these cyclical changes into account [70]. A study considered schizophrenia as an example to demonstrate how a woman’s clinical symptoms can fluctuate throughout the menstrual cycle, leading to changes in medication responses [70], thereby signifying the importance of evaluating the possibility of personalized medication to aid in female reproductive health conditions. As seen in Figure 3, the thickness of the endometrium is influenced by hormonal changes during the menstrual cycle [9,71]. Therefore, the thickness of the endometrium is also impacted by contraceptive use [71]. Certain diseases also establish environmental changes within the UGT due to increased collagen deposition, elevated interstitial pressure, and insufficient vasculature, as is the case with uterine fibroids and tumors [72]. Due to the multifaceted nature of the female UGT, ciliary movement in the fallopian tubes, discarding of the stratum functionalis, uterine contractions, and inconstant blood flow can influence bioavailability and drug distribution [9]. In addition, flow facilitated by bleeding during menstruation can impede the action of locally administered drugs due to dilution or expulsion [73,74]. Hence, the dynamic environment of the female UGT, coupled with the cyclical nature of female hormones and menstruation, poses a major challenge when predicting drug delivery [73,74].

The successful administration of vaginally delivered systems necessitates the bridging of mucosal barriers. Cervicovaginal mucus (CVM) presents an obstacle to drug delivery due to its inherent protective function [75,76]. While this protective function is crucial, it also hinders the penetration of drug molecules and nanoparticles [76]. However, the literature has reported that optimizing the size and surface charge of nanoparticles by adding a coating comprising low-molecular-weight polyethylene glycol (PEG) or utilizing hypotonic formulations can enhance vehicle penetration through the CVM to reach the underlying epithelium [77]. Interestingly researchers expanded on this principle by developing a muco-inert progesterone nanosuspension able to traverse through the CVM while accumulating in the healthy uteri of a pregnant mouse model. This finding is potentially supported by the impact of the uterine first-pass effect, which successfully prohibited PTB in a mouse model [78,79]. Mucin production in the female reproductive tract is not limited to the cervix; thereby, signifying impeded drug delivery throughout the female genital tract [80]. Additionally, enzymes involved in drug metabolism and the expression of transporters throughout UGT tissues predict drug–drug interactions and pharmacokinetic implications [9,81,82].

Drug transporters play a critical role in determining drug exposure and efficacy. Efflux transporters, specifically P-glycoprotein (P-gp or ABCB1) and breast cancer resistance protein (BCRP or ABCG2), serve as significant barriers in the uterus and placenta, preventing fetal exposure to xenobiotics from maternal blood [83]. Notably, the expression of these transporters is subject to hormonal regulation, varying across different stages of the menstrual cycle and during pregnancy. These fluctuations in transporter expression can complicate the prediction of drug performance and drug–drug interactions [9,84]. For instance, the inhibitory effects of progestins on P-gp underscore the necessity of considering co-administered drugs and excipients [9]. In a previous study, it was shown that the phototoxic effects of 5-aminolevulinic acid, a well-established photosensitizer used in photodynamic therapy, were heightened in the endometrial epithelium when a P-gp inhibitor (verapamil) was present [85], thereby signifying the influence of P-gp on drug exposure in endometriosis and suggesting that modifying drug transporters could be an effective strategy for enhancing drug delivery [85]. Metabolizing enzymes also exert a substantial influence on drug exposure, particularly in the metabolism of sex hormones and xenobiotics, which are integral to the pathogenesis of various uterine conditions [86,87]. Strategies aimed at reinstating a hormonal equilibrium should account for polymorphisms in these enzymes, as they may profoundly affect drug efficacy [9]. In tandem with local effects, deviation in hepatic and renal metabolism, paired with altered gastrointestinal transit time, as influenced by the different phases of the menstrual cycle, pregnancy, and menopause, can inflict the disposition of drugs [9,88,89]. Another important factor to consider is that the composition of uterine fluid encompasses an array of compounds and substances that can substantively impact drug distribution [9,51]. Finally, the fate of a drug is contingent on protein binding, as a highly unbound fraction of a drug manifests elevated pharmacological interactions with tissues [90].

Despite drug disposition affecting protein binding, its influence is reduced due to decreased levels of albumin in smaller volume ranges of UGT fluids (i.e., cervicovaginal fluid volume: ±510 µL, and uterine fluid volume: 5–180 µL) compared to plasma volume. However, locally acting drugs can still interact with components of UGT fluid, potentially reducing therapeutic outcomes due to their restrictive exposure [9,51]. Compositional changes in UGT fluids, as inflicted by menstruation and pregnancy, can impact local drug delivery. This impact is attributed to the local dilution of UGT fluids, which can also influence drug ionization, solubility, and protein binding, resulting in unpredictable pharmacokinetic profiles [9]. Protein binding observed in the CVM provides an additional barrier to successful localized vaginal drug delivery [91,92]. Additionally, the vaginal microbiome is less diverse than the microbiome of the UGT. The lower genital tract is mainly populated by a high density of *Lactobacillus* bacteria., whereas the female UGT generally harbors diverse bacteria in a low abundance, such as *Proteobacteria*, *Actinobacteria*, and *Bacteroidetes*, together with *Lactobacillus* [10]. Additionally, dominant microbial species distribution differs between the individual organs of the female UGT. Hence, it is not surprising that increasingly diverse vaginal microbiota, which implies a reduced colonization of the *Lactobacillus* species, predisposes conditions like bacterial vaginosis [93]. Interestingly, research has revealed that specific bacterial species can modulate the pharmacokinetics of vaginally administered drugs by endocytosis together with direct drug binding [93]. Importantly, the described factors also influence the successful and safe targeting of the UGT during pregnancy. Many physiological changes occur to sustain pregnancy such as increasing the plasma volume and body fat, reducing gastric acid secretion, elevating gastric pH, limiting plasma protein binding, nausea, altered metabolizing enzyme activity, accelerated glomerular filtration rate, and modification of drug transporters, which can all contribute to reducing drug exposure due to the altered physiological state. Historically, clinical evaluations in women, and particularly pregnant women, are limited, which further highlights the need to expand scientific insights to improve our understanding of drug pharmacokinetics in the female UGT to allow for tailored drug delivery strategies [9,88].

## 4. Mathematical Models Describing Aspects of Drug Delivery in the Female Genital Tract

When dealing with drug delivery to the female UGT, the duration of the treatment is an important factor to consider as part of providing a tailored, individualized therapy approach. For example, the treatment of endometriosis [94], PCOS [95], postmenopausal hormone treatment, and hormonal products for contraception might all require long-term drug administration that varies from patient to patient [9]. It is, therefore, important to consider patient compliance, as well as drug efficacy and long-term safety, when developing products for drug delivery to the UGT. The vaginal, intrauterine, and intraperitoneal routes have all been explored as options to deliver drugs for the treatment or management of UGT conditions. Of these, the vaginal route of administration might be considered the least invasive by the patient.

Regional drug delivery to the UGT via the vaginal route can be achieved by systemic circulation, progressive diffusion from the cervix and uterus to the endometrium, or the uterine first-pass effect [9,95]. The latter refers to a phenomenon caused by counter-arterial-to-venous perfusion, which leads to higher-than-expected drug concentrations in uterine tissue and low serum concentrations following vaginal administration [95]. An early example of this can be found in a paper by Miles and co-workers, who found that the vaginal administration of progesterone resulted in significantly higher endometrial concentrations when compared to intramuscular administration [96]. More recently, Patel and co-workers investigated the region-selective delivery of paclitaxel to the endometrium via the intra-vaginal route as a means of treating endometrial cancer [97]. Their results suggested that intra-vaginal administration resulted in a high localization of paclitaxel in the endometrium, with low systemic absorption. Specifically, they found that uterine targeting could be increased 20-fold by formulating paclitaxel in nano-sized ultra-deformable vesicles [97]. Moreover, in a study by Zierden et al. [98], different compounds and formulations were tested for their ability to prevent intrauterine inflammation-induced PTB. The results showed that neither systemically administered 17α-hydroxyprogesterone caproate nor vaginally administered progesterone gel were able to sufficiently prevent inflammation-induced PTB. However, vaginally administered muco-inert nanosuspensions containing histone deacetylase inhibitors (trichostatin A and suberoylanilide hydroxamic acid) with progesterone were able to prevent PTB. Pharmacokinetic studies revealed that vaginally administered nanosuspensions lead to increased progesterone concentrations in the distal and proximal myometrial tissue, as well as in the cervix and plasma [98]. Due to the complexity of the anatomy and physiology of the female genital tract, pharmacokinetic behavior prediction by mathematical modeling compared to experimental data can benefit the pharmaceutical industry [99]. Importantly, mathematical models can point to missing components involving biological mechanisms that influence drug delivery when attempting localized drug delivery in the female genital tract [99].

Several excellent mathematical models describing drug delivery via the vaginal route have been developed. Gao and Katz [100] developed a diffusion-based multicompartmental pharmacokinetic (PK) model for the vaginal delivery of tenofovir formulated in a gel. Their system of mass conservation equations is given in Equation (1):

(1a) $$\frac{\partial C_{G}}{\partial t}=D_{G}\frac{\partial^{2}C_{G}}{\partial x^{2}}-k_{D}C_{G}\;\;\mathrm{Gel}$$

(1b) $$\frac{\partial C_{E}}{\partial t}=D_{E}\frac{\partial^{2}C_{E}}{\partial x^{2}}\;\;\mathrm{Epithelium}$$

(1c) $$\frac{\partial C_{S}}{\partial t}=D_{S}\frac{\partial^{2}C_{S}}{\partial x^{2}}-k_{B}C_{S}\;\;\mathrm{Stroma}$$

(1d) $$V_{B}\frac{dC_{B}}{dt}=M_{SB}\left(t\right)-k_{L}C_{B}\;\;\mathrm{Blood}$$

where C is the local drug concentration; D is the diffusion coefficient; V is the volume of distribution; $M_{SB}$ is the volumetric rate of drug entering the blood from the stroma; subscripts G, E, S, and B denote the gel, epithelium, stroma, and blood, respectively; and $k_{D}$, $k_{B}$, and $k_{L}$ are first-order rate constants, denoting the dilution of the gel layer, drug loss to the blood stream, and drug loss from the blood, respectively [100]. The boundary values and initial conditions will not be presented here for the sake of brevity, but can be found in the original paper by Gao and Katz [100]. The predictions made from their pharmacokinetic model were then compared to human biopsy specimens and blood samples published by Schwartz et al. [101]. In general, their model’s predictions agreed with the experimental data, except for some overestimation of the drug concentration in the stroma.

Gao and co-workers later revisited their multicompartmental pharmacokinetic model for the vaginal delivery of tenofovir, this time considering the metabolism of tenofovir to tenofovir diphosphate [102]. Their modified system of mass conservation equations is presented in Equation (2):

(2a) $$\frac{\partial C_{G}}{\partial t}=D_{E}\frac{4w}{V_{G}}\int_{x=0}^{L}{\left.\frac{\partial C_{E}}{\partial y}\right|}_{y=0}dx-k_{D}C_{G}\;\mathrm{Gel}$$

(2b) $$\frac{\partial C_{E}}{\partial t}=D_{E}\left(\frac{\partial^{2}C_{E}}{\partial x^{2}}+\frac{\partial^{2}C_{E}}{\partial y^{2}}\right)-k_{on}\left\{C_{E}\phi_{E}-\frac{C_{DP}}{r}\right\}+k_{off}C_{DP}\;\mathrm{Epithelium}$$

(2c) $$\frac{\partial C_{S}}{\partial t}=D_{S}\left(\frac{\partial^{2}C_{S}}{\partial x^{2}}+\frac{\partial^{2}C_{S}}{\partial y^{2}}\right)-k_{B}C_{S}-k_{on}\left\{C_{S}\phi_{S}-\frac{C_{DP}}{r}\right\}+k_{off}C_{DP}\;\mathrm{Stroma}$$

(2d) $$V_{B}\frac{dC_{B}}{dt}=\int_{0}^{h}\int_{0}^{d}k_{B}C_{S}dxdy-k_{L}C_{B}\;\mathrm{Blood}$$

(2e) $$\frac{\partial C_{DP}}{\partial t}=k_{off}\left\{C_{TFV}\phi -\frac{C_{DP}}{r}\right\}+k_{off}C_{DP}\;\mathrm{Stroma}\mathrm{or}\mathrm{epithelium}$$

where w is the width of the canal, L is the distance from the centre to the edge of the gel, $k_{on}$ is the formation rate of tenofovir diphosphate, $k_{off}$ is the elimination rate of tenofovir diphosphate, $\phi_{E}$ is the volume fraction of epithelial cells, r is the fraction of tenofovir converted to tenofovir diphosphate in the cells, h is the thickness of the epithelium, d is the distance along the canal, and the subscripts TFV and DP refer to tenofovir and tenofovir diphosphate, respectively. All the other symbols have the same meanings as in Equation (1). The boundary and initial conditions are, once again, not presented here but can be found in the original paper [102]. The predictions made by their mass conservation equations were compared to the human biopsy and blood concentration data presented in Schwartz et al. [101]. The predictions made by their compartmental pharmacokinetic model (Equation (2)) were, again, in excellent agreement with the test (human) data. An interesting observation is that Equation (2) slightly underestimated $C_{max}$ for the biopsy data (5.5%) and slightly overestimated it compared to the blood data (19.4%). Conversely, Equation (2) slightly overestimated $C_{24}$ compared to the biopsy data (12.2%) and slightly underestimated it compared to the blood data (2.4%).

Sims and co-workers also developed a compartmental, diffusion-based, pharmacokinetic model for the transport of intravaginally administered nanoparticles [103]. Their set of mass conservation equations considered the barrier posed by the secreted mucus layer of the vaginal epithelium and is presented in Equation (3):

(3a) $$\frac{\partial C_{m}}{\partial t}=D_{m}\nabla^{2}C_{m}-\left(k_{m}+k_{a}+k_{bd}\right)C_{m}\;\mathrm{Mucus}$$

(3b) $$\frac{\partial C_{E}}{\partial t}=D_{E}\nabla^{2}C_{E}-k_{a}C_{E}\;\mathrm{Epithelium}$$

(3c) $$\frac{\partial C_{S}}{\partial t}=D_{S}\nabla^{2}C_{S}-\left(k_{b}+k_{a}+k_{db}\right)C_{S}\;\mathrm{Stroma}$$

where subscript m denotes mucus, and $k_{m}$, $k_{a}$, $k_{b}$, and $k_{bd}$ are first-order rate constants representing nanoparticle clearance from the mucus layer, self-aggregation, clearance into the blood and lymphatic system, and binding and unbinding to the mucin fiber meshwork of the mucus layer, respectively. The rest of the symbols have the same meanings as in Equations (1) and (2). The initial and boundary conditions are, again, not reported here but can be found in the original publication [103]. The predictions made by the model in Equation (3) were compared to experimental transport results obtained from an in vitro vaginal epithelium cell model. The results from their study suggest that the nanoparticle permeation of and accumulation in different layers of vaginal tissue can be controlled by functionalizing the surfaces of the nanoparticles to different degrees with PEG. The model parameters of Equation (3) can be calculated for each PEG-modification density, suggesting that future research can lead to the prediction and customization of patient-specific nanoparticle-based treatments.

At the time of writing this paper, no pharmacokinetic model for the delivery of drugs to the female UGT could be found. However, based on the aforementioned uterine first-pass effect, future research projects could attempt to expand the intravaginal-route pharmacokinetic models described in Equations (1)–(3) to include endometrial tissue, thereby providing mathematical models for drug delivery to the female UGT based on a route of administration that might be associated with high levels of patient compliance.

## 5. The Impact of the Route of Drug Administration

### 5.1. Systemic Drug Delivery

In terms of drug administration in the female genital tract, oral, implantable, injectable, and transdermal drug delivery systems are commonly employed in the management of female UGT disorders [9,104,105]. Disorders such as endometriosis and PCOS are frequently managed via contraceptives to achieve prolonged hormonal regulation. However, the documented safety concerns and compromised efficacy associated with these modalities have fostered an interest in developing innovative drug delivery systems, as well as the exploration of alternative routes of administration [9]. For example, the administration of progesterone via the intramuscular route has resulted in elevated plasma levels but reduced uterine levels in comparison to vaginal administration [9,96]. Systemic routes, specifically intravenous (IV) administration, can facilitate targeted delivery via ligand-functionalized nanoparticles and conjugates [9,106,107]. In contrast, local drug delivery offers the advantage of high drug exposure near the target site, together with circumvention of the hepatic first-pass metabolism [9]. As an example, the initial chemotherapeutic treatment recommended for ovarian cancer is delivered through IV infusion and is often the primary choice for most patients with widespread progression in the peritoneum [108]. Despite the favorable initial treatment response rate, most patients experience a recurrence of the disease within 2 years [108]. The reasoning behind using intraperitoneal chemotherapy is based on the observation that chemotherapeutic drugs administered intravenously result in low concentrations in the peritoneum, regardless of peak serum levels [108]. Furthermore, the peritoneal cavity is recognized as a significantly large area able to enhance exposure of the tumor to the locally administered drugs, thereby reducing the absorption of drugs into the systemic circulation and consequently minimizing its toxicity [108]. Therefore, it is understandable that local drug delivery is considered a potential solution for treating female UGT disorders more effectively.

### 5.2. Local Drug Delivery

Significant advancements have been made in addressing disorders of the female UGT through the vaginal, intrauterine, intraovarian, and intraperitoneal routes of administration [9,43,109,110,111]. For instance, a recent study investigated the novel local application of metformin incorporated into nanoparticles via intraovarian injection. The findings concluded that local intraovarian injection rendered superior treatment outcomes compared to oral treatment in ameliorating hormonal profiles, and immunohistochemical and histopathological features of PCOS in a rat model [111]. This study emphasizes that local drug delivery can facilitate high, sustained local drug concentrations at the anatomical site where pharmacological intervention is required [111], thereby eliminating the need for elevated systemic drug dosages, which also minimizes side effects [111]. It is important when considering the use of drugs like metformin, which is often used but presents some significant side effects like hepatoxicity, pernicious anemia, acute pancreatitis, coagulation alterations, severe hypoglycemia, and, most notoriously, gastrointestinal side-effects known to decrease therapeutic adherence [111].

Despite the advantages presented by local drug delivery, it is imperative to recognize that the pharmacokinetics of drugs differ when exposed to plasma or local cells and tissues. Moreover, different local drug delivery routes impact pharmacokinetics, thereby influencing dosage requirements, safety, and treatment duration [9,109]. The vaginal route of drug administration can be considered to facilitate targeted local delivery to UGT tissues. This can involve diverse pathways like systemic absorption, delivery localized to the cervix and uterus via cumulative drug diffusion into the endometrial lining, and imperatively UGT delivery achieved by the uterine first-pass effect [9,112]. The uterine first-pass effect is a term utilized to describe drug transport mediated by counter currents rendering venous, arterial, and lymphatic system exchange from the lower genital tract to the uterus [92]. The transfer of drugs from the vagina to the uterus theoretically occurs via the exchange of molecules between veins and arteries [52]. This process is facilitated by the dense venous network in the upper vagina to the uterine artery, allowing drugs from the vaginal blood vessels to diffuse into the uterine artery [113]. Blood circulation plays a key role in the uterine first-pass effect, as studies have shown that the concentration of drugs in the uterine artery is significantly higher than in the radial artery [113]. This direct transfer of drugs could pose a challenge for therapies targeting the vagina, as the drugs might be transported away from the intended site of action, hindering the therapeutic drug concentration levels. Since the uterine first-pass effect is limited to the upper third of the vagina, it could significantly impede targeted delivery to the cervix [113]. As a result, larger doses may be required to attain therapeutic drug levels at the cervix [113]. Additionally, drugs can accumulate in the uterus due to increased direct transport from the vagina as facilitated by higher drug concentrations in the lower genital tract. Drug accumulation can potentially cause toxicity challenges, particularly during the administration of chemotherapeutic drugs [113]. Here, nano-based drug delivery systems can offer a particular advantage due to their exceptional targeting capacities, which can counter the need to administer high chemotherapeutic drug dosages [114]. Another important factor to consider during local drug delivery is the enzymatic activity of target tissues, as well as metabolic pathways at the site of drug administration.

While the hepatic first-pass metabolism is known for its high drug-metabolizing capacity, the vagina has relatively low enzymatic activity [9]. Hence, a drug can be administered vaginally rather than orally simply to avoid extensive metabolism of the drug via the hepatic first-pass effect [115]. Interestingly, the vaginal route has facilitated the effective delivery of nanoparticles to the UGT and the draining lymph nodes [115]. Hence, efficacious drug delivery is contingent on the primary drug elimination pathway, which can be enabled by the deliberate drug delivery system design or bypassing elimination pathways due to the site of administration [9,112,113,115,116]. Evidence indicates that local drug administration targeting the upper vaginal channel can circumvent absorption into the portal vein and subsequent first-pass liver metabolism [9]. Moreover, targeted drug delivery to the uterus is achieved by transvaginal administration involving the placement of intrauterine devices for contraceptive purposes. Targeted drug delivery to the uterus also offers a platform to optimize diagnostic evaluations and surgical procedures [9].

The literature reports two distinct barriers to local drug delivery when targeting the uterus and cervix [109]: firstly, the solubility of compounds within the vaginal fluids and, secondly, the permeation of mucus membranes lining the cervicovaginal region. Fortunately, both challenges can be conquered by surface-modified nanoparticles [9]. Researchers have presented other drug delivery alternatives to modify drug delivery in this unique physiological environment [76,77,91,112,113]. However, the incorporation of drugs into nanoparticles is favored as undesirable drug properties can be corrected by adopting traits of the nano-carrier [117]. By modifying drug properties, the half-life of drugs can be extended, resulting in dose reduction and optimized targeting capacity [118,119]. Additionally, literature has reported that localized drug delivery can simplify the delivery of nanotherapeutics via maternal–fetal transfer [12,120,121,122]. This is particularly beneficial where high drug dosages localized at the uterine site can prevent conditions such as PTB [120,123,124]. Ultimately, when deciding on a route of administration, the decision is dictated by the physicochemical properties of the administered drug. This includes properties like solubility in uterine fluids, partitioning into UGT tissues, potential toxicity, size constraints applicable to the drug delivery system, targeted duration of action, affordability, and user-friendliness [9]. Therefore, nano-based drug delivery systems present an attractive approach to drug delivery in the female UGT due to notorious precision and decreased side effects attributed to its small size and targeting capacity [19,20,23,118,125,126,127].

## 6. Nano-Based Drug Delivery Systems

The prefix “nano” originates from the Greek term “nanos”, which refers to “a dwarf”. The official adoption of the prefix “nano” took place in 1947 during the 14th conference of the International Union of Pure and Applied Chemistry (IUPAC), to denote the one-billionth part (10^−9^) of a unit [128]. Nanotechnology entails the deliberate engineering and manipulation of particulate matter to create nano-systems with enhanced functionality, typically within the size range of 1 nm to 100 nm [117]. Nanoparticles, a result of this technological modification, exhibit superior characteristics such as auto-reactive stability and self-reassembly [117]. The adaptability of nanoparticles allows for customization to obtain specific properties, particularly a high surface area in comparison to micron-sized particles [117,129,130]. Nanoscale materials behave distinctively due to surface effects and quantum phenomena, resulting in amplified or novel material properties concerning thermal, mechanical, magnetic, electronic, catalytic, and optical properties. The surface effects of nanomaterials differ from those of micromaterials or bulk materials due to their significantly large surface area, high particle number per mass unit, increased fraction of surface atoms, and surface-situated atoms having fewer direct neighbors. Consequently, these disparities promote alterations in the chemical and physical properties of nanomaterials, for instance, a reduction in the binding energy per atom and subsequent impact on the melting temperature, allowing for customized drug delivery [128]. Importantly, the extent of dispersibility of nanomaterials contributes to generating the desired surface effects to improve the uptake of nanoparticles into biological cells. Additionally, the forceful attractive interactions between nanoparticles can result in aggregation and agglomeration which reduces the surface area and negatively impacts nanoscale drug delivery [131].

Fortunately, an effective prevention of agglomeration can be achieved by elevating the zeta potential, optimizing the hydrophilicity/hydrophobicity, or adjusting the pH and ionic strength of the suspension medium [128]. Within the 1–100 nm range, nanomaterials exhibit size-dependent properties influenced by quantum effects, where the pronounced nature of quantum phenomena in smaller sizes leads to compelling characteristics such as the magnetization of non-magnetic materials at the nanoscale [128,132]. Additionally, quantum effects significantly influence electron affinity, thereby manifesting distinct impacts on the catalytic properties of materials [128], signifying that particle size impacts the therapeutic efficacy and targeting potential of nanomaterials [131].

Nanomaterials can comprise varying sizes and shapes, each possessing distinct drug loading capacity, release kinetics, cell-targeting specificity, and stability that have been reported in the literature [106,131,133]. For the purpose of this review, the use of nanoparticles to aid in female UGT disorders will be considered. The biophysical and chemical attributes of nanoparticles referring to size, geometry, surface charge, chemistry, hydrophobicity, roughness, hardness, and combinability exert significant influence on the efficacy of targeted drug delivery [134]. It is important to note that the size distribution of nanoparticles notably influences drug loading and unloading [131]. Moreover, smaller nanoparticles, characterized by an increased specific surface area, expedite the release of surface-attached drugs [131]. Studies have also indicated that the surface charge of nanoparticles plays a crucial role in their ability to penetrate cells [135]. Positively charged nanoparticles are more likely to be internalized by cells compared to neutrally and negatively charged nanoparticles. This is attributed to the negative charge of cellular membranes [135]. However, with IV administration swift absorption and subsequent protein corona formation on the surface of nanoparticles can complicate nanoparticle-mediated drug delivery. Hence, the literature reports that the interaction observed between nanoparticles and biological cells can render varying results, as evaluated by different studies highlighting that surface charge is the determining factor for protein corona formation [135].

Research also suggests that spherical nanoparticles are more effective at penetrating cell structures compared to their rod-shaped counterparts [131]. An essential determinant influencing nanoparticle drug delivery systems is their effective entry into endosomes, which is contingent upon their surface charge [136]. A recent study demonstrated that engineered uterine exosomes with a negative surface charge improved uterine uptake [137], emphasizing that a positive surface charge can cause rapid opsonization and swift clearance by the reticuloendothelial system, while a neutral or mildly negative zeta potential renders improved compatibility and efficient delivery to uterine target cells [137]. This translocation is contingent upon the surface charge transitioning from negative to positive. Reasons for facilitating surface charge transitioning can be categorized into three groups: (1) maximizing residence and circulation time, (2) targeting specific tissues, and (3) selectively delivering medication to a target tissue [131]. Hence, it is crucial to consider the protein corona when discussing the surface of the nanoparticle, as it stabilizes the particle and ultimately influences the biological response to the nanoparticle [131]. Moreover, the true in vivo particle response depends on surface charge/hydrophobicity and the route of administration [131,138].

The field of nanotechnology is rapidly advancing in terms of scientific research and product development, leading to significant progress across various applications [114,127,130,139]. Its potential application in precision drug delivery holds great promise for medicine [130]. Nano-level engineering allows nanoparticles to act as carriers, transporting drugs to targeted cells or tissues in the body [129]. The modification of surface properties allows nanostructures to selectively target abnormal cells while limiting their impact on healthy cells [11]. For the purpose of regenerative medicine, nanomaterials can potentially serve as scaffolds utilized in tissue engineering or act as carriers for signaling molecules to facilitate the repair and regeneration of injured tissues [11,140]. Although the field of nanomedicine is still emerging, its potential applications, including the diagnosis and treatment of female UGT disorders, show promise for significant improvement. The application of early and accurate female UGT disorder diagnostic practices, facilitated by nanotechnology, has been extensively reported in the literature [118,141,142,143,144]. Therefore, this review focuses on selected nanoparticles and their specialized applications in addressing female UGT disorders. Additionally, safety considerations and prospects are discussed to pave the way for the development of future nanotechnologies.

## 7. Nanoparticles

**Figure 4:**
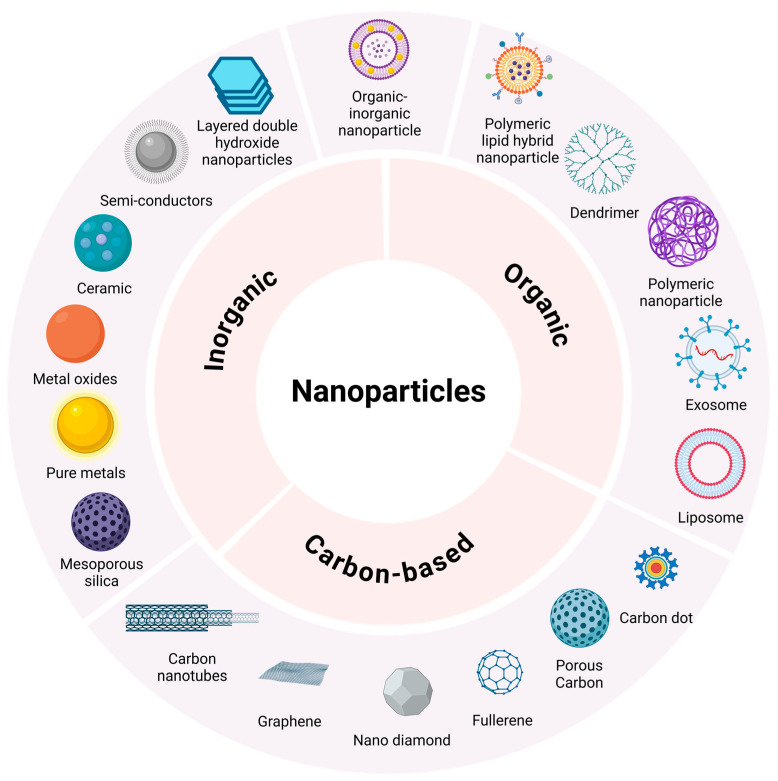
Visual representation of nanoparticle classification. Created in BioRender. Van staden, D. (2024) https://BioRender.com/y17n846 [9,11,106,128,149,150,161,166].

Nanoparticles are objects with external dimensions ranging within the nanoscale, implying that no severe discrepancies exist between the longest and shortest axis of these minute objects [128]. However, upon observing drastic dimensional differences where the shortest axis is at least three times shorter than the longest axis, these nano-objects are instead referred to as nanofibers or nanoplates [128]. Interestingly, nanoparticles can transpire into numerous sizes, shapes, and structures able to harbor conical, cylindrical, hollow-core spiral, spherical, tubular, and irregular forms [145]. Typically, nanoparticles fall within the size range of 1 to 100 nm, and are instead referred to as atom clusters if smaller than 1 nm [128]. The modification of physicochemical properties of nanoparticles, such as size, shape, lipophilicity, functional groups added to nanoparticle surfaces, surface coating and surface charge, allows for the prediction of the capacity of nanoparticles when interacting with biological systems [146]. Importantly, the physicochemical properties of nanoparticles can also predict the suitability for a specific application. In terms of the female reproductive system, nanoparticles can be employed for diagnostic purposes or aid in therapeutic intervention based on their physicochemical properties [11]. As an example, nanoparticles can be used as imaging agents to detect disease and define disease progression due to inherent physicochemical properties like fluorescent emission that is influenced by particle size [146]. In contrast, therapeutic approaches involving nanoparticles depend on physicochemical properties such as surface charge to enable the crossing of barriers to drug delivery to improve the targeting capacity of nanoparticles [56,146]. On the other hand, the shape and size of nanoparticles can also influence targeting capacity, which is imperative when aiming to decrease side effects and reduce dosing intervals [146]. Importantly, forces impacting the release of drugs from nanoparticles should be carefully considered during nanoparticle development to find a balance between drug carrier accumulation and drug release behavior when desiring targeted drug delivery [147]. The nanoparticle class, as well as the excipients used during the production of nanoparticles, can drastically influence release kinetics [147]. As an example, the erosion of a nanoparticle chitosan matrix upon exposure to an acidic environment can be linked to enhanced electrostatic attraction existing between water molecules and protonated amine groups, which can mitigate rapid drug release [148]. Therefore, it is essential to consider each nanoparticle class when discussing forces that can impact drug release from nanoparticles. Table 2 and Figure 4 present a summary of the different classes of nanoparticles and includes examples of nanoparticles that are employed to diagnose and treat female UGT conditions.

**Table 2:** Composition-based classification of nanoparticles.

Nanoparticle Classification	Nanoparticle Sub-Classification	Example	Reference
Inorganic	Pure metals	Al*, Cd*, Co*, Au*, Ag*, Zn*	[128,149,150]
	Metal oxides	Fe_2_O_3_*, Al_2_O_3_*, ZnO*, TiO_2_*	[128,149]
	Ceramic	ZrO_2_*, SiO_2_*	[128,149]
	Semi-conductors Layered double-hydroxides Silica	ZnS*, CdS* Mg^2+^*, Zn^2+^*, Cu^2+^*, Al^3+^* MPSNs*	[128,151] [152] [153]
Organic	Lipid	Liposomes, Niosomes, Transferosomes, Exosomes, SLNs*, SEDDSs*, Nano-emulsions	[9,11,128,150] [154] [154] [9,11] [155] [156] [9]
	Polymeric	Polymeric nanoparticles, Polymeric micelles, Dendrimers	[106] [106] [106]
	Protein-based	Ferritin nanocages, Silk protein fibroin carrier, Human serum albumin, Gliadin carrier, Gelatin carrier, Legumin carrier	[157] [157] [157] [157] [157] [157]
Organic hybrid	Lipid and polymeric	Polymersome, Lipomer, Polyplex	[158] [159] [158]
	Lipid and protein	Lipoprotein carriers	[151]
	Protein and polymeric	Protein-loaded polymeric nanoparticles	[160]
Carbon-based	Carbon nanotubes	Single-wall, Double-wall, Multi-wall, Unzipped multi-wall	[161] [161] [161] [162]
	Graphene	Nanoribbons, Quantum dots	[161] [161]
	Nano-diamonds	Detonation nano-diamonds, Fluorescent nano-diamonds	[161] [161]
	Fullerene	Endohedral metal fullerene, Exohedral metal fullerene, Substituted fullerene	[151,161] [151,161] [163]
	Porous carbon	Microporous (<2 nm), Mesoporous (2–50 nm), Macro porous (>50 nm), Mixed porous carbon	[161] [161] [161] [161]
	Carbon dots	Graphene dot, Carbon nano-dot, Polymer dot	[161] [161] [164]
Hybrid nanoparticles	Organic–inorganic	Gold nanoparticle liposomes	[106,165]

*Al: aluminum, Cd: cadmium, Co: cobalt, Au: gold, Ag: silver, Zn: zinc, Fe_2_O_3_: iron oxide, Al_2_O_3_: aluminum oxide, ZnO: zinc oxide, TiO_2_: titanium dioxide, ZrO_2_: zirconium dioxide, SiO_2_: silicon dioxide, ZnS: zinc sulfide, CdS: cadmium sulfide, Mg^2+^: magnesium ion, Zn^2+^: zinc ion, Cu^2+^: copper ion, Al^3+^: aluminum ion, MPSNs: mesoporous silica nanoparticles, SLNs: solid lipid nanoparticles, SEDDSs: self-emulsifying drug delivery systems.

As demonstrated by Table 2 and Figure 4, nanoparticles are a vast, constantly expanding drug delivery and diagnostic field, rendering infinite combinations and possibilities. To add to the variability of nanoparticles, these carriers can display a single- or multi-crystal solid nature, along with amorphous structures, and may also be present in loose or agglomerated configurations [128]. Moreover, nanoparticles can be uniform or comprise multiple layers. These layers are described as a surface, shell, and core layer, where the core layer represents the central portion of the nanoparticle. The surface layer generally encompasses various minute molecules, surfactants, metal ions, or polymers [151], whereas the shell layer is composed of a material exhibiting clear chemical distinction from the core layer. Importantly, nanoparticles can also comprise porous nanomaterials known for superior drug loading capacity and vast applications in nano-based imaging [167]. Therefore, nanoparticle classification can be based on their composition, categorizing them into four groups: inorganic, organic, carbon-based, and hybrid nanoparticles composed of a combination of organic–inorganic materials [128,151].

### 7.1. Inorganic Nanoparticles

Nanoparticles have been explored as vehicles for delivering, sensing, and imaging purposes [150]. Inorganic nanoparticles, with unique characteristics such as adjustable morphology, easy functionalization, desirable physiological stability, and special physicochemical properties like optical, electrical, acoustic, and magnetic natures, hold great promise in nanomedicine [149]. The two most notorious types of inorganic nanoparticles are metal-based and metal oxide-based nanoparticles [114]. Metal-based nanoparticles are produced from metals modified to the nanoscale by employing a bottom–up or top–down approach [150]. On the other hand, metal oxide-based nanoparticles are manufactured via the modification of corresponding metal-based nanoparticles [150]. The primary objective of synthesizing metal oxide-based nanoparticles is to enhance their reactivity and efficiency [168]. Therefore, inorganic nanomaterials exhibiting superior performance characteristics are increasingly favored in clinical applications of female reproductive disorders. Examples include optimized antitumor therapy and improved delivery of antibacterial agents [126,130,169,170].

#### 7.1.1. Pure Metal Nanoparticles

Research has investigated the use of nanoparticles made from metals such as gold, silver, titanium, and platinum in addressing cervical cancer [170]. Gold nanoparticles are considered a promising approach for delivering genes and drugs without causing toxicity, offering stability, selectivity, and controlled drug release within cells [170]. Moreover, gold nanoparticles have shown potential for cancer screening, imaging, and delivering chemotherapeutic drugs for cervical cancer [170,171,172]. A study reported, generating gold nanoparticles encapsulating doxorubicin (DOX) while capped with resveratrol which induced apoptosis in HeLa and CaSki cell lines, suggesting a promising approach for treating cervical cancer [173]. Additionally, these particles also reduce toxicity in normal cells while increasing toxicity and promoting necrosis in HeLa cells when combined with curcumin, implying potential for bioimaging and anticancer applications for cervical cancer [170,174]. Pure metal nanomaterials also exhibit intriguing magnetic properties and potential applications as magnetic nanoparticles [175]. For example, in the work conducted by Shalaby and co-workers, a novel programmable drug delivery system employed magnetic nanoparticles to selectively target uterine fibroid cells [176]. Furthermore, research has reported localized, non-surgical adenovirus-based treatment for uterine fibroids, by combining viral-based gene delivery with nanotechnology to enhance drug-targeting precision [11,176]. Interestingly, metal oxide nanoparticles can be produced from pure metal nanoparticles to optimize drug targeting in the female UGT.

#### 7.1.2. Metal Oxide Nanoparticles

In terms of metal oxide nanoparticles, zinc oxide (ZnO) nanoparticles have been considered for treating female UGT cancers [130]. The minute size of nano-ZnO enables efficient absorption within the human body [177]. In comparison to other metal oxide nanoparticles, ZnO nanoparticles present a cost-effective and relatively non-toxic alternative, rendering them suitable for various medicinal purposes, including antimicrobial, anti-diabetic, anti-inflammatory, anti-aging, wound healing, and bio-imaging applications [178,179]. Moreover, the high biocompatibility of ZnO nanoparticles permits their application in therapeutic settings for antibacterial, antifungal, antiviral, and anticancer properties [178]. While several inorganic metal oxides such as titanium dioxide (TiO_2_), copper oxide, and ZnO have undergone production and continued investigation, some sources describe ZnO nanoparticles to stand out owing to their cost efficiency, safety, and ease of preparation [178]. Furthermore, any consumable agent intended for diverse disease treatments should ideally be non-toxic, non-reactive with food or the container, palatable or tasteless, and devoid of an unpleasant odor. ZnO nanoparticles effectively meet all these criteria [178].

However, limited knowledge exists on the toxicity implications of ZnO nanoparticles, particularly toxic effects involving female germ cells [180,181]. Germ cell differentiation is described as a complex biological process that can be negatively impacted by environmental insults rendering altered fertility [181]. Therefore, it is important to mention the results of a recent study investigating the impact of ZnO nanoparticles on mouse ovarian germ cells in an in vitro system [181]. This study investigated the toxic effects of ZnO nanoparticles on germ cells by visual inspection with light microscopy, assessing cell proliferation, the quantification of reactive oxygen species (ROS) levels, general cytotoxicity studies, and analysis of gene expression. Their results revealed that ZnO nanoparticles produce cytotoxic effects directly related to the exposure time and concentration of ZnO nanoparticles introduced to mouse ovarian germ cells [181]. On the other hand, the green synthesis of ZnO nanoparticles can provide an alternative to improve toxicity profiles of ZnO nanoparticles [182,183]. For instance, a recent study performed the synthesis of ZnO nanoparticles with the aqueous leaf extract of *Ipomoea aquatica*, demonstrating positive outcomes regarding anti-microbial, hemocompatibility, anti-inflammatory attributes and in vitro cytotoxicity [182]. Therefore, the safety of ZnO nanoparticles employed to aid in female reproductive system disorders should still be established.

#### 7.1.3. Semi-Conductor Nanoparticles

On the other hand, metal oxide semiconductor-based biosensors have become integral to the advancements in biotechnologies and bioelectronics due to their exceptional properties, which collectively enhance efficiency and applicability [184]. In the field of female reproductive health, the precise determination of follicle stimulating hormone (FSH) levels holds significant importance for diagnosing endocrine diseases and aiding in female infertility treatment [139]. Recently, Pareek and co-workers [185] designed a nano-molecular-imprinted polymer (MIP)-based electrochemical sensor for FSH detection in PCOS cases utilizing nickel cobaltite (NiCo_2_O_4_) and reduced graphene oxide (rGO). NiCo_2_O_4_ is known as a quality p-type semiconductor and rGO behaves like semi-metal or semiconductor due to its electrical conductivity which can be adjusted by regulating its oxygen content [186,187]. Hence, to prepare this platform, the indium tin oxide electrode surface was modified with the application of NiCo_2_O_4_/rGO nanomaterials. Next, the surface was covered with FSH-MIP. Hereafter, the electrochemical sensor-based platform quantified FSH in spiked blood samples. The relative standard deviations obtained were 96.28, 98.79, 90, and 94.15% for different concentrations (1 μM, 10 nM, 100 pM, 0.1 pM), therefore demonstrating the reliability of the nano MIP-based assay [185].

#### 7.1.4. Ceramic Nanoparticles

As the final inorganic nanoparticle subtype discussed in this review, ceramic nanoparticles represent a category of inorganic nanomaterials extensively employed in a variety of applications, including (photo)catalysis, bioimaging, drug delivery, and wound healing [188]. These nanoparticles are synthesized in different compositions, such as zirconia, silicon nitride/carbide, and silica/alumina titania, in various morphologies and forms (i.e., amorphous, polycrystalline, dense, hollow, or porous) through successive cooling and heating methods [188]. Ceramic nanoparticles exhibit diverse bioactive properties, such as antimicrobial activity and tissue regeneration, rendering them suitable for wound healing [188]. Within this category, well-established members like nanohydroxyapatite and calcium triphosphate find applications in drug delivery and bone tissue engineering [188]. In terms of female reproductive health, an investigative study focused on the translocation of zirconia nanoparticles (ZrO_2_ nanoparticles, 16 nm), known for their relative biocompatibility, to fetal brains within three exposure models of pregnant mice. Model 1 entailed the oral exposure of nanoparticles before the maturation of the maternal blood–placental barrier (BPB); Model 2 involved exposures after the development of the BBB but before the full maturation of the fetal blood–brain barrier (BBB); and Model 3 encompassed exposures after the full development of both the maternal BPB and the fetal BBB. The results indicated a 55% higher translocation of ZrO_2_ nanoparticles into fetal brains in Model 2 and a 96% higher translocation in Model 1 compared to that in Model 3 following the oral exposure of nanoparticles (50 mg/kg) to pregnant mice. Consequently, it was inferred that nanoparticles are capable of traversing multiple biological barriers, and the nanotoxicity to the fetus is notably contingent on the stage of pregnancy and fetal development or the maturity of the biological barriers. Notably, oral exposure to nanoparticles during pregnancy poses potential risks to fetal brain development, particularly in the early stages of pregnancy. Therefore, the impact of nanoparticles on fetal development should still be established and their potential risk in pregnancy should be further investigated [122]. Apart from the impact on fetal development, the importance of producing biocompatible nanoparticles should be highlighted to ensure sustainable treatment options.

#### 7.1.5. Layered Double-Hydroxides

The two-dimensional structure of layered double-hydroxides (LDHs) deems this type of nanoparticle excellent therapeutic carriers [152]. Interestingly, LDHs can harbor nearly any stable element presented in the periodic table due to small metal cations framing the spaces between the layers of LDHs where metals can be incorporated in a complex or anionic state [152]. This is attributed to an elevated surface-to-volume ratio and the potential of harboring large molecules. Additionally, LDHs are known for simplified production procedures, affordability, decreased risk of causing toxic effects, and encapsulation of intercalating agents [189]. However, research revealed that LDHs are generally degraded when introduced to an acidic environment implying that rapid drug release can occur when LDHs enter the stomach [152,189]. This warrants an innovative dosage form design to fully capitalize on the carrier potential of LDHs. The literature reports the successful development of a mucoadhesive LDH carrier demonstrating biocompatibility when studied in three divergent cell lines, including cervical (HeLa) cells [189]. This study describes a highly relevant approach for potentially facilitating localized drug delivery via vaginal administration to gain access to the uterus via the uterine first-pass effect [72,95,189]. This can be a valuable strategy to locally deliver antimicrobial agents and hormones to the UGT [189]. Moreover, a recent study reported LDH nanoparticles as promising candidates for providing embryo protection while treating pregnant cancer patients [190]. Remarkably, LDH nanoparticles comprising etoposide exhibited the inhibition of tumor growth without any impact on embryonic development in mouse embryonic stem cells [190]. These results are attributed to a positive LDH surface charge that enables targeted distribution into tumor mitochondria potentially via selective regulation of the cysteinyl aspartate-specific proteinase-3-gasdermin E pathway [190].

#### 7.1.6. Mesoporous Silica Nanoparticles

The honeycomb-resembling porous structure of mesoporous silica allows for the loading of functional and therapeutic agents via diffusion mechanisms rendering superior drug loading capacity [191]. Precision drug delivery can be facilitated by mesoporous silica harboring magnetic materials or the modification of the mesoporous silica surface without cytotoxic implications [192]. Moreover, drug release from kinetics from mesoporous silica can be influenced by modifying the diameter of mesoporous silica pores. In terms of shortcomings, leakage from mesoporous silica-based systems before reaching the target site has been reported [192]. Additionally, hemolysis inflicted by interactions between groups such as surface silanol present on the surface of mesoporous silica and phospholipid groups found in the membranes of red blood cells can be a drawback of utilizing mesoporous silica as a nanoparticle final material [192]. A publication by Mazzotta and co-workers provided an in-depth consideration of mesoporous silica as a starting architecture instead of a final material to mitigate drug release [191].

Mesoporous silica nanoparticles (MSNPs) present a promising option for drug delivery because of their unique mobile composition of matter-41 (MCM-41) structure [193]. This structure allows for the incorporation of various macro- and micro-molecules, and adding divergent functional groups internally or externally [130]. MSNPs can be modified with specific biomarkers to create targeted drug delivery systems [130]. The successful binding and internalization of MSNPs into cancer cells rely on their effective penetration and localization within the tumor. MSNPs possess distinctive characteristics, including excellent biodistribution, biocompatibility, and a large surface area with a high drug loading efficiency for both imaging and treatment purposes [130]. To achieve targeted drug delivery, two strategies are employed with MSNPs. Firstly, different moieties are introduced to the pore outlets through covalent bonds, which are subsequently released at the pathogenic site due to the breakdown of bonds triggered by external or internal stimuli. Secondly, polymeric or lipid shells can be added to the surface of the MSNPs, causing the drug to be released upon disruption of the polymeric layer [193].

As an example of the application of MSNPs in female reproductive health, cannabinoids (CBDs) show potential as anti-cancer agents for epithelial ovarian carcinomas [194]. This can be attributed to the aggressive stage of the disease resulting in the overexpression of CBD receptors in ovarian cancer [130]. Due to the hallow nature of MSNPs, these structures present advanced potential regarding drug loading. Moreover, their attributes such as mesoporous structure, chemical characteristics, and functional surface emphasize attractive qualities required for the generation of specialized delivery systems [195]. Moreover, human cells and cancerous cells are capable of MSNP uptake via endocytosis pathways [130]. However, CBD, being an unstable molecule with low aqueous solubility, limits its administration via the parenteral route [110]. Hence, enhanced nanoencapsulation can improve the stability of CBD and enable its effective use, as is the case with MSNPs [130].

### 7.2. Organic Nanoparticles

#### 7.2.1. Lipid-Based Nanoparticles

**Figure 5:**
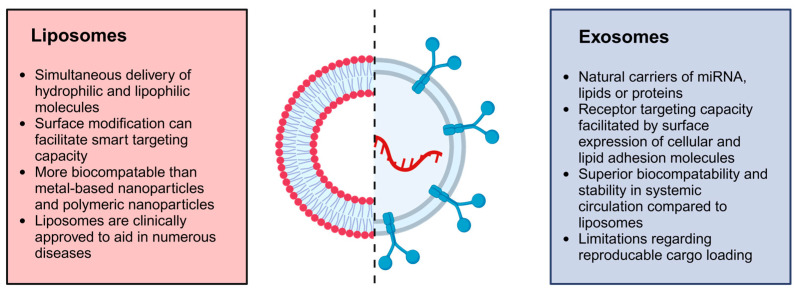
Visual comparison of the most important differences between liposomes and exosomes as lipid-based nanocarriers. Created in BioRender. Van staden, D. (2024) https://BioRender.com/a41p751 [202].

Lipid-based nanoparticles are considered promising candidates for drug delivery due to their versatile properties that allow for the encapsulation of drugs with divergent physicochemical attributes. Moreover, lipid-based nanoparticles are perceived as superiorly biocompatible if compared to other classes of nanoparticles [196,197,198]. Importantly, well-established techniques are employed to manufacture lipid-based nanoparticles, indicating their practical potential for extensive clinical applications [106]. Despite the advantages presented by lipid-based nanoparticles, challenges remain regarding their potential toxicity and clearance from the body, which requires refinement for successful clinical application [106]. Presented as small droplets or vesicles confined by phospholipidic layers, lipid-based nanoparticles are generally produced to form bilayer structures [106,199]. Hence, tailoring lipid-based nanoparticle properties involves careful selection of excipients to exercise precise control over release rates of incorporated drugs and tissue-targeting capacity to aid in conditions like ovarian cancer [200,201]. With regards to lipid-based nanoparticles, this review briefly considers liposomes, one of the most established lipid nanocarriers, along with exosomes, a trending lipid nanocarrier that exhibits increased biocompatibility and high therapeutic versatility compared to traditional lipid-based nano-systems. Figure 5 summarizes the key differences between liposomes and exosomes.

##### Liposomes

Among lipid-based nanocarriers, liposomes provide abundant formulation options due to simplified preparation methods, relative chemical versatility, and adequate size control [203]. Ranging in the diameter size of 80–200 nm, liposomes are described as vesicular self-assembled nanocarriers predominantly produced with modifiable combinations of phospholipid and cholesterol [106,203]. Liposomes are distinguished from micellular lipid-based aggregates due to bilayer membrane formation resulting in the separation of an internal aqueous lumen from an external aqueous phase. Additionally, the vesicular morphology of liposomes allows for the incorporation of hydrophilic drugs in both aqueous regions. Moreover, liposomes provide superb potential in active and passive therapeutic targeting due to simplified size control practices, chemical versatility, and modification of mechanical attributes [204]. The multifaceted applications of liposomes are evident when considering the literature. As an example, results presented from a multicenter, open-label, non-inferiority randomized controlled trial, with trial number ChiCTR2000038555, compared the safety and toxicity of liposomes encapsulating paclitaxel to the generally used combination of paclitaxel and carboplatin as an established first-line therapy to aid in epithelial ovarian cancer [205]. The findings of this trial affirm the non-inferiority of paclitaxel liposomes when weighed against the combination of paclitaxel and carboplatin specifically considering therapeutic efficacy, accompanied by an enhanced safety profile [205]. Moreover, research has revealed liposomes as beneficial when combined with ultrasonic therapy due to beneficial effects, such as superior vascular permeability, reduced pressure caused by interstitial tumor fluids, ameliorated perfusion and oxygenation, and stimulation of the immune system, without precipitating toxic effects in the patient [206].

##### Exosomes

On the other hand, exosomes, as a natural nano-sized member of extracellular vesicles (30–150 nm), are produced during the fusion of endosomes with the plasma membrane. Exosomes have garnered significant attention from the research community over the past decade [9]. Microscopy images reveal that exosomes are spherically shaped with a lipid bilayer akin to liposomes and a negative charge under physiological conditions [9]. These extracellular vesicles are formidable facilitators of cell–cell communication [207,208,209]. Hence, exosomes inherently carry biological cargoes (i.e., micro-ribonucleic acid (miRNA), lipids, or proteins) to recipient cells from mother cells to facilitate cell–cell communication [210]. Exosomes are considered ideal for targeted drug delivery due to their exceptional properties such as high biocompatibility, small size facilitating deep entry of tissues, and stability in blood [211]. Typically, exosomes are collected from the supernatant of target cells that were subjected to repeated centrifugation and ultracentrifugation [212,213]. Exosomes originating from mesenchymal stem cells (MSCs) are not processed prior to use or subjected to bio cargo loading [9,214]. Several active and passive loading strategies like loading small interfering RNA (siRNA) and miRNA via electrophoresis, incubation with drugs or permeabilizers, sonication, click chemistry, and freeze–thaw techniques have been reported [9,214].

Apart from techniques used to isolate exosomes, the crucial contribution of exosomes in the diagnosis and management of female reproductive disorders has been extensively reported. Examples include reproductive system cancers, endometriosis, infertility, intrauterine adhesions, and preeclampsia [215,216,217,218,219,220,221]. Regarding their role in disease therapy, studies have shown that exosomes derived from miRNA 214 (miR-214)-enriched ectopic endometrial stromal cells reduced fibrosis in mouse endometrial peritoneal implants, indicating their potential in disease management [9,222]. Additionally, exosomes derived from menstrual MSCs aided in the improved development of follicles together with stabilizing estrogen cyclicity and serum levels in a rat model evaluating premature ovarian failure [223]. However, despite the benefits of exosome-based treatment compared to MSCs, concerns about immunosuppression, tumorigenesis, and practical limitations of large-scale manufacturing have been raised [9,224]. Furthermore, exosomes lack multipotency and differentiation potential, potentially necessitating larger and more frequent doses due to their low circulation and tissue half-life [9]. Therefore, despite their promising attributes, further research is warranted to address these limitations before fully capitalizing on the therapeutic potential of exosomes.

#### 7.2.2. Polymeric Nanoparticles

Similar to lipid-based nanoparticles, polymer-based nanoparticles provide flexibility in delivering drugs by enclosing various therapeutic agents, including small molecules, proteins, and nucleic acids [106]. Due to the availability and advanced development of polymers, these versatile building blocks of polymeric nanoparticles provide the simplified modification of nanoparticles to govern surface properties, release rate of drugs, and superior adaptability for targeted therapeutics [225]. In contrast, polymeric nanoparticles also demonstrate limitations such as challenges in accurately controlling the release of active substances accompanied by potential toxic effects and immune-mediated reactions to foreign entities like polymeric nanoparticles [106,225]. Importantly, the long-term stability of polymers is a crucial factor to consider as the literature reports unpredictable behavior of polymer-based nanomaterials upon exposure to different pH environments and the dynamic activity of enzymes which can alter the release kinetics of incorporated drugs [106,226,227]. Hence, scientists are constantly investigating and reporting novel approaches to conquer the limitations of polymeric nanoparticles. For example, it is documented that pH-responsive polymeric nanoparticles can facilitate targeted therapeutic outcomes [106]. These findings are based on the discovery that polymers can be pH-sensitive which allows for management of the drug release rate to sustain therapeutic drug concentrations at localized target sites [228]. This approach not only enhances drug efficacy, but also decreases potential adverse effects on healthy tissues. Nevertheless, maintaining consistent and targeted drug release is challenging because pH levels can significantly vary in tumor microenvironments and even within the same tumor [106]. Importantly, pH-responsive polymers might not be sensitive to moderate pH shifts within the biological environment, causing complications such as inappropriate drug release at non-targeted anatomic regions [106]. These complications can be addressed by innovative methods to amplify polymer responsiveness to altered pH environments. The literature recommends combining stimulus-responsive entities with polymeric nanoparticles, allowing for the control of drug release kinetics [229,230]. Alternatively, research reports the potential of targeting mechanisms mediated by ligand–receptor interactions or targeting facilitated by magnetism leading to optimization of precision drug delivery [106]. Another approach used to advance polymer-based nanoparticle drug delivery is to prolong residing of drugs in the systemic circulation and increase the accumulation of various drug delivery systems in tumor cells. The typical approach involves surface conjugation with biocompatible organic materials such as PEG, PEG-derivatives, red cell membranes, etc. [106,231]. Biopolymers such as hyaluronic acid (HA), a natural polysaccharide with a negative charge, are abundantly present in the extracellular matrix and possess desirable biocompatibility, nontoxicity, and biodegradability [232,233]. Additionally, HA can enhance accumulation at the tumor site and actively target certain tumor cells by specifically binding to the overexpressed CD44 receptors on the surface of many types of cancer cells [234,235]. Moreover, hyaluronidase, present in the tumor microenvironment, can break down HA chains, exposing the wrapped drug delivery system, thereby enabling a range of cancer treatments with biocompatible vehicles [236]. In a recent study, a one-pot synthesis process was refined to stabilize the lipophilic model drug, D-α-tocopherol succinate (α-TOS), in zeolitic imidazolate framework-8 (ZIF-8) compounds (referred to as α-TOS@ZIF-8), which was subsequently coated with a HA shell, rendering the HA/α-TOS@ZIF-8 nanoplatform [236]. The HA shell provided a smart switch function governing precise tumor targeting and accumulation mediated by the CD44 pathway. In short, the biocompatible HA-based shell was deteriorated by hyaluronidase activity in tumor cells, uncovering protected α-TOS@ZIF-8, followed by the acid-mediated decomposition of ZIF-8 in the tumor microenvironment, thereby facilitating α-TOS release at a specified tumor site. This study signifies a novel contribution to improving treatment efficiency, which aids in targeting cancers affecting the female UGT [236].

#### 7.2.3. Protein-Based Nanoparticles

The literature describes proteins as notorious biopolymers comprising linear peptide chains capable of folding into complex three-dimensional structures [237]. Proteins play a crucial role in almost all biological processes, such as the transportation of small molecules, cellular signaling, and providing structural support within cells. Additionally, proteins present high biocompatibility due to their well-defined structure and widely reported specific interactions involving target receptors [238]. Therefore, proteins can be considered therapeutic entities, as well as highly effective carriers able to transport various drugs, resulting in enhanced treatment efficacy [239]. For example, recent research reported the employment of leuprolide to aid in endometriosis via prolonged retention in the bloodstream [240]. Therefore, utilizing proteins to form nano-sized particles artificially can help overcome many limitations of individual proteins, such as prolonging circulation time, increasing cellular uptake, and enabling spatial proximity for cascade reactions, making it a subject extensively discussed in the literature [237,239,241,242].

Protein-based nanoparticles can be generated via supramolecular assembly, the aggregation of denatured proteins, cross-linking in emulsions, assembly of conjugates with polymers and coacervation of charged polymers and proteins [237,243]. Each method used to manufacture protein-based nanoparticles has specific advantages and disadvantages depending on the intended use [237,244]. For instance, while employing denatured proteins to form aggregates is a common approach for drug delivery, the loss of the protein’s three-dimensional structure restricts its use in other applications [237]. Similarly, although different methods for protein nanoparticle preparation can enhance cellular uptake or circulation times, this action can also significantly influence the immune response to protein-based nanoparticles [244], highlighting that the uncontrolled aggregation or severe degradation of proteins can provoke the immune system, potentially resulting in severe allergic reactions [244]. A potential strategy to overcome the shortcomings of lipid-, polymeric-, and protein-based nanoparticles is to develop organic hybrid nanoparticles by harnessing the benefits of more than one class of organic nanomaterial.

### 7.3. Organic Hybrid Nanoparticles

For instance, incorporating the biomimetic properties of liposomes and the structural advantages of polymers has led to significant progress in producing multifunctional lipid–polymer hybrid nanoparticles (LPHNs), offering great potential for targeted and efficient drug delivery [245]. The therapeutic efficacy of these LPHNs varies depending on the characteristics of the different components used during formulation of organic-hybrid nanoparticles [245,246]. The LPHN core–shell is structurally composed of distinct elements. It includes a biodegradable polymer component that enhances the entrapment efficiency of poorly water-soluble therapeutics [245,247,248]. Additionally, lipid mono- or bi-layers facilitate structural stabilization, which is paired with selective ligand cross-linking designed to protect against immune system recognition and enhance the time residing in systemic circulation [245]. Hence, it is crucial to carefully select appropriate types of lipids and polymers that complement the incorporated drug(s) while considering the physiological conditions of the target site in order to achieve the site-specific localization of both hydrophobic and hydrophilic drugs [246]. In applications of hybrid delivery systems, it is essential to prolong circulation time by evading detection and clearance by the reticuloendothelial system [245]. Strategies can involve active targeting via the modification of nanoparticle surfaces with multiple extracellular ligands [245,249,250]. These grafting moieties can be attached either to the distal end of polymers or directly incorporated into the lipoidal membrane to customize drug delivery [245].

Despite the customization potential of organic–hybrid nanoparticles, challenges arise during the upscaling of LPHNs due to various factors which currently restrict protein-based nanoparticle applications in biomedicine, particularly in monitoring, bioimaging, and drug delivery [251]. To overcome these challenges, bio-modifications have shown significant promise in producing consistent LPHNs for targeted drug delivery, diagnostics, and preventive applications [245,252]. LPHNs have been engineered to offer numerous benefits, including stability, high cargo loading capacity, improved biocompatibility, controlled release, extended drug half-lives, and enhanced therapeutic effectiveness while mitigating their limitations [245]. Interestingly, research has revealed that hybrid systems such as liposomes and chitosan particles have successfully addressed these shortcomings, with mucoadhesive nanocarriers showing particular promise in the treatment of cervical cancer [253,254]. This is supported by a study reporting that a liposome–chitosan nanocarrier system enhanced the permeability of curcumin considerably, signifying superior formulation compared to traditional systems for vaginal administration, and thereby facilitating cell entry for drug delivery against cervical cancer [253,254]. Since lipids are the building blocks of cellular membranes, the lipophilic component of organic–hybrid systems reduce the concentration of synthetic polymer inclusion that could potentially impact biocompatibility of nano-carriers

### 7.4. Carbon-Based Nanoparticles

Carbon, as an essential building block of deoxyribonucleic acid (DNA), presents an intriguing alternative to organic nanomaterials [161]. The unique electron configuration (1s^2^, 2s^2^, 2p^2^) of carbon allows it to exist in a wide range of crystalline and non-crystalline forms. Carbon is an incredibly versatile element, characterized by diverse allotropes and structures. The specific type of allotrope is determined by carbon’s hybridization (sp^3^, sp^2^, and sp^1^) and its bonding involving other atoms [161]. For instance, diamonds are created through sp^3^ hybridization, while graphite is formed through sp^2^ hybridization [255]. This versatility of carbon finds applications in various technologies, such as drug delivery systems and the production of synthetic materials [161,255,256,257]. Carbon’s capability to form bonds with nearly all elements underlines its widespread utility. Hence, carbon nanomaterials possess exceptional physicochemical properties, such as thermal, optical, electrical, mechanical, and structural diversity, in contrast to other nanoparticles [258]. The unique characteristics of the hollow cylindrical graphitic sheets, known as carbon nanotubes, provide enhanced flexibility, strength, and electrical conductivity for interacting with biological entities, which is advantageous for medical diagnosis and treatment purposes [258]. Conversely, the planar graphitic sheets, or graphene, can be readily modified on the surface using different functional groups; thus, enabling specific and selective detection of numerous biological components [259]. Furthermore, the large surface area, chemical purity, and free π-electrons provided by graphene hallmarks an ideal choice for drug delivery [259]. The ability to effectively interact with various fluorescent dyes, drugs, and other biomaterials deems them a valuable tool for in vivo imaging, diagnosis, and cancer treatment [258]. Important factors such as enzymatic degradation, surface modification, biological interactions, and bio-corona have been thoroughly considered in the literature to aid in creating carbon-based drug delivery systems for efficient drug delivery [260]. Additionally, carbon-based target-specific and release-controlled drug delivery techniques are utilized to enhance treatment effectiveness [161]. The scientific community is actively focusing on the development of new production methods for carbon-based nanoparticles to make their production more attractive to the industrial sector [256]. Due to the nano size and diverse physical properties of carbon-based nanoparticles, distinct biological interactions necessitating comprehensive preclinical toxicity studies before advancing to clinical applications are required [257].

#### 7.4.1. Carbon Nanotubes

Carbon nanotubes (CNTs) are primarily tubular or cylindrical, varying in length, diameter, layers, and chirality. Their unique blend of elasticity, strength, and rigidity provides various appealing drug delivery properties [261]. CNTs can be classified based on their structure as single, double, multi-walled, or functionalized [259,262]. Various methods such as chemical vapor deposition, arc discharge, laser ablation, and high-pressure carbon monoxide disproportionation are used for synthesizing CNTs [263]. This carbon-based nanotechnology possesses distinctive characteristics rendering CNTs highly favorable drug carriers. Characteristics include ultrahigh surface area, enhanced cellular uptake, high drug loading capacity, and effective transportation ability [259]. Additionally, CNTs exhibit good electrical and thermal conductivity, excellent mechanical and electrical properties, inert nature, and the ability to bind to organic and inorganic compounds, implying suitability for many applications [259]. When examining the efficiency of drug loading based on their sizes, it has been observed that short CNTs have an enhanced drug loading efficiency than longer ones. However, after 72 h of incubation, longer CNTs displayed higher efficiency [259]. The literature also considers CNTs as valuable tools for gene delivery due to their excellent surface properties [264,265]. The aspect ratio of CNTs is linked to excellent cell penetration capability, while anisotropic conductivity/semi-conductivity along the CNT axis is ideal for integration with nervous and muscular tissues [266]. Moreover, the large surface area maximizes CNT’s ability to interact with biological matter, and the hollow interior promises vast capacity for drug delivery [259]. Furthermore, surface functionalization can adjust solubility and biological recognition [267]. Despite the potential of CNTs to deliver drugs, genes, and other biomedically essential materials, their use is restricted mainly due to unpredictable toxicity profiles [267]. However, the reactive nature of the surface of CNTs allows for attaching guest molecules (i.e., drugs, siRNA, and diagnostics) to increase their biocompatibility [267]. Therefore, requirements for CNTs can be adjusted by substituting guest molecules to improve accumulation in tumor cells while maximizing the targeting of cancerous cells [264,265,266,267,268].

A useful example is employing folate receptor α (FRα), which is abundant in cancer cells while eliciting a low expression in healthy cells, to facilitate the uptake of carriers into cancerous cells, therefore serving as the main target for modification due to the high demand for folate in cancer cells [259,269]. This is especially important when targeting ovarian cancer cells which exhibit elevated expression of FRα compared to other cancer types. The approval of mirvetuximab soravtansine (MIRV) in 2022 as the first antibody–drug conjugate utilizing FRα as targeted cancer treatment affirms the accuracy of capitalizing on this drug targeting strategy. As an approved therapy, MIRV treatment can aid in FRα-positive, platinum-resistant epithelial cancers affecting the female reproductive tract [269]. Interestingly, the folate receptor mediates the internalization of the nanoparticles through caveolae-mediated endocytosis [259]. Therefore, the addition of biological components, chemical entities, or bioactive compounds can neutralize challenges involving solubility and toxicity-related reactions [259]. Moreover, adding the aforementioned components can enable the functionalization of CNTs via surface binding that improves drug targeting and diagnostic precision. The functionalization of CNTs exploits both covalent and noncovalent bonds, where a covalent modification alters its electronic structure and non-covalent bonding refers to general physical interaction [259,268].

The detailed interactions between nanoparticles and biological systems are crucial to understanding the large-scale production of diverse nanomaterials. It is particularly important to uncover the structural details of these interactions and explore any potentially harmful effects of nanoparticles. Estrogen receptors (ERs) are imperative for maintaining ovarian granulosa cell differentiation, facilitating the development and growth of follicles and oocytes, and ovulation function [270]. Therefore, any abnormalities involving estrogen, its receptors, and estradiol-synthesis-related enzymes significantly impact clinical reproductive endocrine diseases, such as PCOS and endometriosis [270]. It is important to consider that the binding of carbon nanotubes to ERs can potentially trigger reproductive toxicity associated with carbon nanotubes [271]. A recent study provided insight into the toxic reproductive repercussions by evaluating single-walled carbon nanotubes (SWCNT). As a model nanomaterial, SWCNT was subjected to dynamic molecular modelling, in vivo studies, and spectroscopy-based assays [271]. Results clarified information gaps related to binding interactions between binding domains for ligands of ER alpha (ERα) [271]. Importantly, fluorescence-based assays and simulations of molecular dynamics both verify binding interactions between SWCNT and ERα [271]. Experimental results implied that conformational changes were facilitated at a tertiary structure level as primarily enforced via hydrophobic forces [271]. In vivo data highlighted the potential estrogen-disrupting effects upon SWCNT exposure enhancing expression of ERα from 26.43 pg/mL to 259.01 pg/mL, hence raising a potential red flag for health risks associated with SWCNT binding to ERα [271]. Fortunately, other carbon-based nanoparticles like graphene are considered more biocompatible.

#### 7.4.2. Graphene

Graphene is described as a remarkably lightweight compound harboring an individual layer of carbon atoms resembling a honeycomb lattice configuration. The literature boasts its elastic nature, contrasted with the knowledge that graphene exceeds the strength of steel by 200 times [272]. A recent study reported the generation of a material comprising a graphene-based nanocomposite (GBN) rendering an advanced improvement in the functionality of graphene [273]. Fascinatingly, the newly generated graphene-based material exhibited superior safety and biocompatibility profiles when coming into contact with living tissues, making it suitable for surgical applications [273]. Currently, this GBN material is being investigated for use in heart valves, breast implants, and tendons, among other applications. This research aimed to use the GBN material to create a surgical membrane for treating pelvic organ prolapse (POP) [273]. POP negatively impacts the life quality of nearly half the global female population [274]. POP is attributed to a weakened pelvic floor resulting in pelvic organ protrusion into the vaginal canal. Currently, therapeutic options involve lifestyle-mediated changes or inserting a temporary silicone-based pessary [273]. However, pessary insertion demands replacement procedures that can cause secondary discomfort. Surgically, the repair of native tissues or augmentation procedures via polyproline mesh modification can be considered [273]. Unfortunately, due to safety discrepancies, polyproline implant use is prohibited in most North American countries, Australia, and the United Kingdom. Hence, the newly generated GBN can provide a promising and potentially permanent solution to aid in POP and related conditions [273].

In terms of drug delivery, graphene has become a widely used nanocarrier for drug delivery applications because of its two-dimensional structure, exceptional strength, large surface area, ease of surface modifications, and strong biological compatibility [272,275]. The use of nanoscale graphene oxide (GO) for drug delivery was first reported by the Dai research group in 2008 [276,277]. An effective nano-carrier was developed by incorporating the anticancer drug DOX into PEG functionalized GO to target tumor cells in a controlled environment [275]. A novel binate drug loaded carrier, was generated from anionic graphene oxide/cationic polyethyleneimine/polyanionic dextran sulfate (GO/PEI/DS), via layer-by-layer self-assembly. This nano-carrier was designed to aid in anticancer therapy via skin-mediated drug delivery [275]. DOX, the selected drug, was loaded onto folic acid-conjugated GO. On the other hand, methotrexate (MTX) was loaded onto dextran sulfate. Cytotoxic assessments were performed by utilizing a MCF-7 breast cancer cell line. Findings confirmed the combined effect of DOX and MTX rendered reduced cancerous cell growth while preserving cell viability of >87.65% in a normal L929 cell lines [275]. In vivo pharmacokinetic studies comparing oral and skin-mediated routes of administration revealed that skin-mediated drug delivery of the newly developed nano-system achieved sustained and prolonged systemic drug concentrations [275]. To compare, oral administration exhibited a mean systemic drug residence time of 83.98 ± 3.71 h, whereas skin-mediated administration obtained a value of 149.62 ± 6.11 h. Interestingly, the novel drug loaded system demonstrated pH-mediated release profiles for both incorporated drugs facilitating controlled release and sustained dosing profiles following skin-mediated drug delivery [275]. Therefore, emphasizing the versatile applications of graphene which can be linked to the versatility of carbon and other carbon-based nanoparticles like nano diamonds (NDs).

#### 7.4.3. Nano-Diamonds

NDs visually resemble diamonds due to their tetrahedrally bonded carbon atom configuration imitating a three-dimensional cube-shaped lattice [161]. These nano-carriers are categorized into detonation NDs (DNDs) and fluorescent NDs (FNDs) based on the synthesis process and size. DNDs, typically around 5 nm in size, are produced through shockwaves using explosives like trinitrotoluene and hexogen, while FNDs exhibit a wider size distribution and are generated under conditions involving high pressure and elevated temperatures [278]. NDs are known for readily binding to various ligands, chemical compounds, and drug molecules, as attributed to their flexibility in sp^2^/sp^3^ bonds [161]. Notably, NDs with nitrogen-vacancy defect centers emit fluorescence within the 550–800 nm range. This distinctive property has led to their use in creating fluorescent probes for single-particle tracking in complex environments, making NDs a valuable tool in bio-imaging research [161]. Additionally, NDs are utilized for delivering drugs with low aqueous solubility, such as G9a inhibitors, through conjugation [279]. This composite is suitable for IV administration, and the drug release is pH-responsive. With functionalization, this class demonstrates superior biocompatibility compared to CNTs [161]. Interest in NDs is steadily increasing in the theragnostic field [280]. Furthermore, the application of NDs in medical sciences are endless as these nanostructures enable biodegradable orthopedic surgery, the generation of scaffolds, tissue engineering possibilities, and the delivery of genetic materials [278,279,280,281,282].

In recent years, FNDs have made significant advancements as a new addition to nanocarbon technology. The surface area of FNDs can present crystallographic defects comprising color centers that provide superior characteristics when compared to other fluorochromes (i.e., fullerenes, quantum dots, and nanotubes) [282]. Additionally, due to the inherent properties of FNDs, their potential in diagnostics is highlighted by the inclusion of FNDs in the development of bio-imaging technology, nanoscale magnetic structure imaging, and nanometric temperature sensing [282,283,284]. The structure of FNDs encompasses specific surface point defects, among which the negatively charged nitrogen-vacancy center is the most extensively studied color center [282].

Recent research involving ND application in female reproductive health involved the development and characterization of unmodified and surface-modified (-COOH and -NH_2_) NDs for their ability to load efavirenz followed by assessing their cytotoxicity in vitro [285]. Findings indicate that the unmodified ND-conjugated drug formulation exhibited an enhanced capacity for drug loading together with limited toxicity when compared to surface-modified NDs. Additionally, it is documented that unmodified-ND conjugated drug delivery vehicles were evaluated according to their drug dissolution behavior and BBB migration allowing for the prediction of therapeutic efficacy [285]. These biological characterizations serve as the basis for further in vivo studies on the pharmacokinetics and pharmacodynamics of ND-based HIV drug delivery. HIV Type 1 (HIV-1) continues to be a significant global cause of mortality [285,286]. Although current combination antiretroviral therapy has considerably improved HIV-1-related pathology the literature highlights the need to facilitate successful delivery of therapeutic agents to reservoir organs of HIV. The central nervous system is an example of a HIV reservoir organ and drug delivery to this organ is impeded by BBB transmigration [285,287]. Therefore, the application of NDs to aid in female reproductive health has sparked innovative developments due to the superior biocompatibility of NDs signifying them as more efficient drug carriers when compared to other carbon-based materials [285,288].

#### 7.4.4. Fullerene

Compared to NDs, fullerenes can also be described as efficient drug carriers. Fullerenes have a spherical shape, comprised of 60 carbon atoms, 12 pentagons with single C5–C5 bonds, and 20 hexagons with double C5–C6 bonds [289]. Additionally, fullerenes contain fused rings and conjugated bonds with a hybridization of sp^2^ and sp^3^, with an average bond length of 0.145 nm for single bonds and 0.141 nm for double bonds [289]. Their truncated icosahedral symmetry ensures uniformity in the environment of every carbon atom. Most notoriously fullerene C_60_, with the smallest cage structure, exhibits high reactivity and stability by adhering to the isolated pentagon rule, where each pentagon is surrounded by 5 hexagons [289]. Due to poor electron delocalization, fullerenes are electron deficient, making them effective antioxidants widely used in cancer therapy [289]. The hydrophobic nature of fullerenes results in low solubility in polar solvents but high solubility in organic solvents such as benzene, chloroform, and toluene [289,290]. To enhance their solubility in polar solvents, fullerenes can be derivatized with polar groups [291]. The capacity of fullerenes to be subjected to numerous chemical reactions as electron acceptors is complemented by their ability to encapsulate ionic species due to the inertness of fullerene [289].

The unique properties of fullerenes, including high hydrophobicity, cohesivity between molecules, photoactivity, reactivity, and electron acceptance and release capabilities, enable diverse chemical transformations and structural modifications for extensive biomedical applications [292,293]. For instance, a study exploring fullerene application in the field of female reproductive health found that ultrasound acoustic pressure waves can penetrate biological tissues more deeply than light and induce light emission known as sonoluminescence [292]. This led to the consideration of using fullerenes as an alternative energy source to excite photosensitizers. Pristine C_60_ fullerene (C_60_), an excellent photosensitizer, was investigated for its potential in cancer sonodynamic therapy in combination with low-intensity ultrasound treatment [292]. The analysis indicated that C_60_ accumulated in human cervix carcinoma HeLa cells reached its maximum at 24 h (800 ± 66 ng/106 cells), with half of the extranuclear C_60_ localized within mitochondria. Cell-based assays demonstrated the effectiveness of the C_60_ nanostructure’s excitation with 1 MHz ultrasound, resulting in a significant proapoptotic sono-toxic effect on HeLa cells [292]. The ability of C_60_ to induce apoptosis in carcinoma cells after sono-excitation with ultrasound highlights that fullerene can provide a promising novel approach to aid in female cancer treatment [292].

#### 7.4.5. Porous Carbon

Porous carbons present an interesting alternative to other carbon-based nanoparticles due to simplified synthesis procedures and the added advantage of its porous structure to enhance drug loading capacity [294]. Moreover, these porous structures can harbor either organic or inorganic materials inside porous passages or on external surfaces allowing for tailored drug delivery approaches to improve therapeutic targeting capacity [294]. Mesoporous silica is an example of such a material, providing a starting nanoarchitecture platform alone or in combination with porous carbon for the development of advanced nano drug delivery vehicles [153,191,295,296,297]. The application of porous carbon and mesoporous carbon silica nanoparticles in disorders affecting the female reproductive system was limited at the time of writing this review. However, the potential of utilizing these systems individually or in combination with materials such as mesoporous silica to aid in female reproductive systems disorders cannot be ignored. At this stage, the application of MSNPs in female genital tract disorders can be described as the pioneer for combining porous carbon and mesoporous silica due to the successful application of MSNPs in female UGT disorders.

In terms of female UGT disorders, a study has reported the development of a highly sensitive multi-unit integrated electrochemical biosensor array as facilitated by nitrogen-doped mesoporous carbon (NMC) [298]. NMC presents improved conductivity, modifiable pore size and enhanced affinity for detecting catalytic enzymatic activity. These favorable attributes improved the sensitive and rapid quantification of carbohydrate antigen 125 in human blood serum. Thereby, providing a platform for future research to improve biomarker detection in biological analysis to simplify early and accurate diagnosis of diseases such as ovarian cancer [298].

#### 7.4.6. Carbon Dots

In contrast to the porous nature of MSNPs, carbon dots are small spherical/quasi-spherical carbon nanoparticles of typically less than 10 nm in size, primarily made up of carbon, hydrogen, oxygen, and nitrogen [299]. Carbon dots have many easily modifiable surface groups such as epoxy, carboxyl, and hydroxyl, which contribute to their good water solubility [282,299,300]. Generally, carbon dots are produced from raw materials like citric acid, chitosan, glucose, and sucrose [299]. As more researchers seek eco-friendly synthesis methods, biomass, traditional Chinese medicine, and plants are increasingly employed to explore synthetic raw materials to reduce environmental impact [301]. Quercetin, an inexpensive and easily extractable compound found in various foods and medicinal plants, is a crucial component of the flavonoid family with medicinal properties [299]. Moreover, scientists are considering quercetin as a green raw material for carbon dots synthesis due to its abundance and eco-friendliness [299]. The synthesis of carbon dots can generally be categorized into “top-down” and “bottom-up” approaches [302]. The hydrothermal method, known for its simplicity, wide carbon source applicability, and cost-effectiveness, is commonly employed in laboratories for large-scale production of these carbon-based nanoparticles [299,303]. Owing to their desirable luminescent properties, stability, low cytotoxicity, easy preparation, accessibility of raw materials, and ease of storage, carbon dots find widespread application in bio-sensing, disease diagnosis, biological imaging, and drug delivery [164,299,303,304,305].

Researchers are shifting their focus to the fluorescent capacity of carbon dots for substance analysis and detection [299]. When an analyte is introduced to a carbon dots solution, the fluorescence intensity of the carbon dots is either enhanced or quenched, establishing a linear relationship for substance detection [306,307]. Many fluorescence analysis methods based on carbon dots have been applied to detect drugs, metal ions, and pesticide residues [299]. Recently, a novel study produced nitrogen carbon dots with yellow-green fluorescence via a hydrothermal method employing quercetin and o-phenylenediamine as raw materials [299]. These nitrogen carbon dots exhibited good water solubility, high dispersibility, and optical properties, with a fluorescence quantum yield of 6.45%. A Cell Counting Kit-8 (CCK-8) assay indicated their low cytotoxicity and favorable biocompatibility. The synthesized nitrogen carbon dots demonstrated selective and efficient quenching by oxytocin, with a detection limit of 0.0196 IU/mL, and were used to detect oxytocin in oxytocin injections, with a recovery rate of 98.8–103.8% [299]. The quenching mechanism of the system was identified as static quenching [299]. Notably, the fluorescence of the nitrogen carbon dots was sensitive to pH variations, and stability varied at different pH levels, necessitating careful pH adjustment during the detection process [299]. This study sets an example for rapid and sensitive drug quantification. Oxytocin induces strong uterine contractions and is commonly used for labor induction [308]. Therefore, proper dosage accuracy and determination of active pharmaceutical ingredients in oxytocin preparations are essential to ensure clinical safety during oxytocin administration and to ensure improved female reproductive health outcomes [299].

### 7.5. Organic–Inorganic Nanoparticles

Organic–inorganic nanoparticles represent a promising approach to mitigate concerns regarding organic nanoparticle accumulation by harnessing the collective advantages of organic and inorganic materials [166]. Nevertheless, further investigation is imperative to comprehensively discern potential interactions and enduring implications of these hybrid nanoparticles within the human body. The immune response to hybrid nanoparticles is contingent upon factors such as size, shape, and surface properties. Although organic nanoparticles are generally acknowledged as biocompatible, the introduction of inorganic constituents may incite an immune response, potentially instigating inflammation or other unfavorable effects [309,310]. Hence, an extensive exploration of the immune response to these hybrid nanoparticles is essential to ascertain their safety profile before widespread integration in medical applications [166]. Research revealed that smaller hybrid nanoparticles with spherical morphology and smooth surfaces increased the probability of circumventing the immune system and were deemed biocompatible [311]. Conversely, larger nanoparticles characterized by irregular shapes and rough surfaces elicited a more robust immune response, leading to inflammation and plausible health hazards [106,312]. Hence, highlighting the pivotal significance of meticulously evaluating the physical attributes of nanoparticles to ensure their safety in medical settings [106].

The literature reports the potential of combining organic-inorganic composites to fabricate nanoparticles, harboring attributes such as magnetism and optical properties, to facilitate targeted drug delivery and controlled release [11]. Moreover, considering drug delivery, organic–inorganic hybrid nanocarriers provide the option of loading two bioactive agents with distinct physicochemical properties into a single delivery system [313]. Importantly, the co-delivery of drugs can significantly decrease the concentrations of certain drugs required to establish anticancer activity [313,314]. A recent study delineated the fabrication of hybrid particles encompassing organic–inorganic composite structures, each providing distinct physical domains [313]. The organic segment is fabricated from the prevalent biodegradable polymer poly(lactic-co-glycolic) acid, while the inorganic matrix comprises organosilica [313]. It was suggested that the organic–inorganic vehicle composition can contribute to versatility in co-loading drugs and nitric oxide (NO), along with favorable attributes of modulated drug release and enhanced drug stability [313]. Preceding studies have demonstrated that including NO donors with conventional chemotherapeutic agents augments anticancer efficacy; thus, laying the groundwork for the development of NO-based combinational anticancer therapy [315,316]. This study verified the viability of co-delivering camptothecin and NO using organic–inorganic composite nanocarriers [313]. The development of these hybrid nanocarriers demonstrated the feasibility of combining organic-inorganic composites to benefit targeted cancer therapy which can aid in targeting cancers of the female UGT [313].

## 8. Safety of Nano Drug Delivery Systems

The extensive utilization of nanoparticles has given rise to apprehensions regarding their potential toxicity within the human body, particularly in relation to the male and female reproductive systems and fetal health [317]. Apprehensions are based on the minute dimensions of nanoparticles, their superior capacity to cross biological barriers, biocompatibility, and their potential to breach the placental barrier, thereby causing damage and toxicity to the fetus and exerting potentially toxic effects on the female reproductive system [151,225,317,318]. Due to the limited follicle count in females, it is imperative to identify factors contributing to oocyte damage and reduced fertility [319]. Furthermore, the growth and renewal of female reproductive organs, including the uterus and ovaries, are under the regulation of hormones [11]. Moreover, disruption of hormonal regulation may lead to fetal anomalies [317]. This consideration is highlighted by studies reporting that environmental nanoparticle contaminants have demonstrated toxic effects on the reproductive system and embryonic growth [318,320].

**Figure 6:**
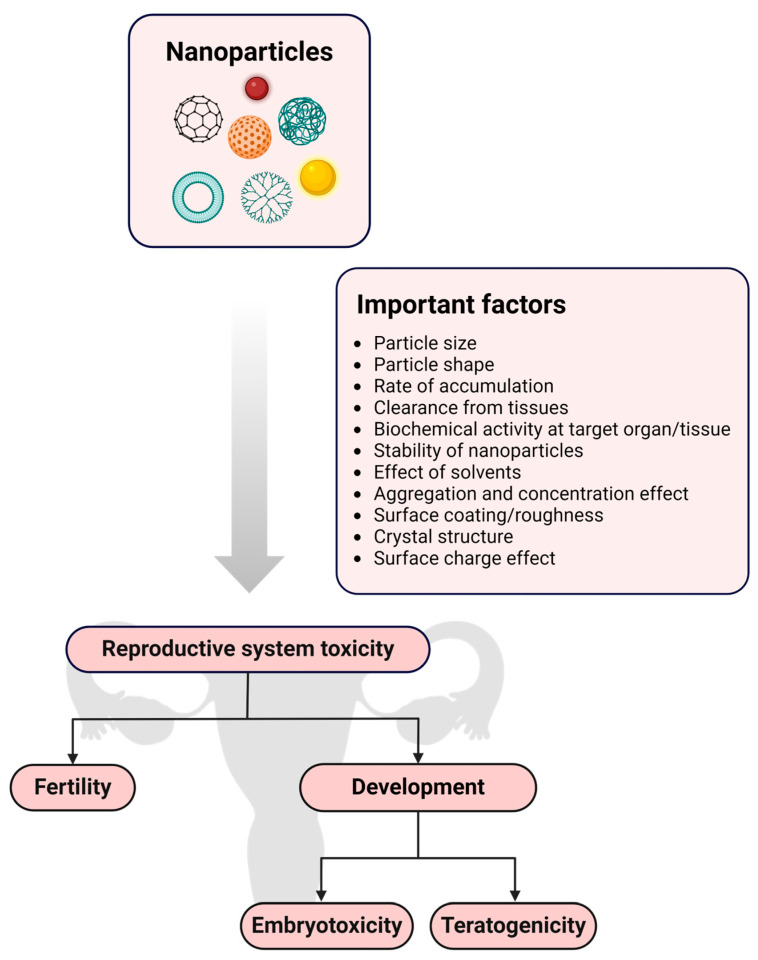
Illustration of important factors involved in nanoparticle-related toxicity in the female reproductive tract. Created in BioRender. Van staden, D. (2024) https://BioRender.com/s68j683 [317].

The literature states the imperatives of establishing the short- and long-term toxicity of reproductive organs exposed to nanoparticles [317]. As attributed to the small size of nanoparticles, surface-to-mass ratio, enhanced stability, variations in biocompatibility and excretion from the body, predicting the behavior of nanoparticles within biological systems is inherently challenging [151,317,318,321,322]. Importantly, the toxicity of nanoparticles is influenced by their rate of tissue accumulation and clearance [317]. Additionally, stable nanoparticles are known to exhibit heightened toxicity upon prolonged exposure [317]. However, particle size emerges as a reliable determinant of toxicity, with larger particles demonstrating increased accumulation relative to smaller ones, particularly within specific regions of the ovaries [317]. As seen in Figure 6, nanoparticle-related toxicity is impacted by different factors.

The morphology of nanoparticles also plays a crucial role in their interaction with biological systems (as seen in Figure 6) [317]. The potential toxicities of individual nanoparticles were considered in previous sections of this review. Generally, spherical nanoparticles can effectively target cell membranes during processes such as phagocytosis and endocytosis, facilitating their entry into cells [317]. In comparison to non-spherical nanoparticles, spherical nanoparticles demonstrate simpler and quicker endocytosis, leading to an easier entry into the bloodstream and subsequent biological effects [323]. Furthermore, the surface charge of nanoparticles significantly influences cytotoxicity and various aspects of nanoparticle behavior within the body, including selective uptake, BBB integrity, colloidal behavior, and plasma protein binding [317]. Moreover, positively charged nanoparticles tend to exhibit higher cellular uptake, while negatively charged nanoparticles can induce hemolysis and platelet aggregation [317]. In addition to shape and surface charge, the crystalline structure and chemical composition of nanoparticles are important determinants of their toxicity [324]. Research has revealed that different dimensions and particle sizes of silver and copper nanoparticles could be linked to the extent of toxicity in zebrafish, dolphin, and algae species, whereas TiO_2_ nanoparticles with similar dimensions rendered no toxic effects, demonstrating the impact of crystalline structure and nanoparticle constituents in toxicity [317,325,326]. Importantly, the dissolution of nanoparticles in various mediums can also alter their crystalline structure, influencing toxicity profiles which can present drug delivery challenges due to the shifting pH environment of the female reproductive tract, which is also influenced by conditions such as pregnancy [67]. On the other hand, surface coatings of nanoparticles have the capacity to modify their physicochemical properties, affecting nanoparticle distribution, accumulation, toxicity, and pharmacokinetics within biological systems [317,318,324]. Accumulation patterns of nanoparticles are dependent on factors such as surface charge, composition, and size, with significant implications for nanoparticle toxicity profiles [322,324]. Overall, the medium and solvent conditions can significantly impact the dispersion and agglomeration of nanoparticles, thereby influencing particle size and ultimately toxicity within biological systems [317].

Considering the environmental effect of nanoparticles is imperative as these minute entities have high accumulation potential, implying long-term toxicity effects [327]. Therefore, it is guaranteed that nanoparticles entering the environment will influence living organisms, and the composition of soil and water ultimately invading the lifeline of societies [327]. Establishing regulatory frameworks to navigate practices involving nanotechnology in medical applications is thoroughly discussed in a recent publication by Luo and co-workers [11]. However, the integration of nanomaterials in clinical translation is pivotal for advancing nanomedicine, yet it is confronted with multifaceted challenges [328]. The main challenge is the standardization of nanomaterial production followed by quality control regulations to find international consensus on the reproducibility and implied safety of nano-based drug delivery systems [329,330]. Additionally, the lack of standardized toxicity evaluation methods obstructs a comprehensive understanding of potential risks associated with nanomaterials [11]. Therefore, it is understandable that an ethical concern with nanotechnology revolves around safety. These safety concerns should receive holistic investigation regarding the health of patients subjected to nano-based therapeutics, medical practitioners working with nanotechnology to detect diseases and researchers developing nanomaterials to aid in reproductive health [11,331]. Hence, as emphasized by restrictions on assisted reproductive technologies employment of nano-based drug delivery systems calls for further research and validation concerning long-term safety and feasibility.

## 9. Discussion

After considering the intricate anatomic structures and dynamic physiological environment of the female genital tract, it is evident that both localized and systemic drug delivery present challenges [9,11]. One of the most notorious barriers to efficacious drug delivery is the mucosal barrier guarding the female reproductive system. As discussed, this mucosal barrier differs from mucus membranes lining the gastrointestinal and respiratory tracts. Thus, emphasizing the importance of targeted drug delivery approaches and tailorable functionalization of therapeutics to avoid trapping and degradation of drugs [136,170,178,312]. Nanoparticles present an attractive solution to drug delivery challenges in the female genital tract due to their tailorable targeting capacity, as provided by endless possible excipient combinations and innovative formulation strategies to overcome restrictions to efficacious drug delivery while reducing side effects [123,245,332].

Importantly, disorders affecting the female UGT can impact normal physiological systems in an already dynamic physiological environment and therefore further complicate localized drug delivery. As an example, endometriosis, an inflammatory, hormone-dependent condition, necessitates targeted drug delivery strategies to preserve the normal endometrial tissue to improve the quality of life and fertility outcomes in endometriosis patients [94]. On the other hand, nanomaterials hold exceptional promise in the early diagnosis of female reproductive system diseases which can aid in avoiding disease progression [11,48,125,126,178]. Moreover, the literature reports techniques to modify the surface and surface charge of nanoparticles leading to improved targeting capacity [77,135,146,148,152,246]. As a future recommendation, nanoparticles that exhibit rapid drug release upon exposure to an acidic environment, which can impede oral drug delivery, can be seen as favorable when aiming to facilitate rapid drug release in the acidic vaginal environment to mediate drug delivery to the female UGT via the uterine first-pass effect. However, the preparation of nanoparticles is complex due to expensive production costs, lack of standardized regulations and the technological advancements needed to allow large-scale reproducibility [11,333,334]. Self-assembly, nanoparticle self-assembly, and microfluidic technology are reported as the main innovative techniques utilized to produce nanoparticles [335,336,337,338,339]. However, despite the remarkable advancements presented by these production methods upscaling remains problematic.

In terms of easy upscaling possibilities, investigating the potential application of self-nano-emulsifying drug delivery systems (SNEDDSs) in female reproductive health provides a promising approach to simplify the production of nano-based drug delivery systems [340,341]. Among various approaches, self-emulsifying drug delivery systems (SEDDSs) have emerged as a promising strategy to overcome mucosal barriers [56]. SEDDSs are formed when combining an oily phase, comprising oil(s), a surfactant(s), and a co-surfactant that emulsifies spontaneously when introduced to an aqueous environment [342]. This typically results in the formation of nano-sized droplets [342,343,344,345]. Research has reported equipping a spontaneous nano-emulsion with thiol moieties facilitating interaction with mucus, destructing disulfide bonds within the mucus network which facilitated the successful crossing of mucus membranes [56]. Moreover, researchers have produced zeta-potential-changing SEDDS via a flip–flop mechanism designed to cross mucus and epithelial membranes [346,347]. This technique allows for the rapid permeation of hydrophilic and negatively charged nanocarriers through the mucus barrier before facilitating interaction between a hydrophobic and less negatively, or preferably positively charged nanocarrier to enhance cellular uptake [347], thereby signifying zeta-potential-changing SEDDSs as a promising strategy when considering future applications for facilitating the transport of drugs across mucus membranes guarding the female genital tract.

Apart from innovative formulation techniques and drug delivery strategies, the development of a pharmacokinetic model to predict drug delivery in the female UGT can be highly relevant during the pre-formulation phase of nanoparticles destined for female UGT targeting. A mathematical model capable of considering the variable thickness of endometrial tissue, as influenced by UGT disorders and the menstrual cycle can facilitate accurate prediction of drug delivery in the complex environment presented by the uterus, thereby simplifying the development of personalized nano-based drug delivery systems aiding in female UGT disorders.

## 10. Conclusions

Nanotechnology has enormous potential to aid in female UGT disorders. This review highlights the application and the advancement in nanoparticle use in the field of female reproductive health. Targeted drug delivery in the female UGT is challenging due to the dynamic physiological environment and intricate anatomical structures. Therefore, nanoparticles are continuously explored for their specialized targeting capacity, ability to incorporate numerous drugs in reduced drug concentrations, and potential for personalized medication. However, the short- and long-term toxic effects of nanomaterials on the female reproductive system require further investigation, especially when considering the multi-generational impact of accumulated toxicity in organs such as the uterus. Innovative and simplified upscaling possibilities should also be explored to fully capitalize on the potential of nano-based drug delivery systems. In conclusion, the rapidly evolving field of nanotechnology can give rise to personalized and precise strategies to aid in female UGT disorders.

## Figures and Tables

**Table 1:** Physiological variations during the menstrual cycle that can impact drug pharmacokinetics [69].

Physiological System	Phase of Menstrual Cycle	Potential Pharmacokinetic Impact
**Renal system**		
Creatine clearance	↑ late luteal phase/	↑ renal clearance
Glomerular filtration rate	↓ menstrual phase	↓ renal clearance
Vasopressin and aldosterone	↑ luteal phase	
Renin activity	↑ luteal phase	↓ renal clearance
Urinary kallikrein excretion	↑ luteal phase	
**Gastrointestinal system**		
Esophageal emptying	↓ luteal phase	↓ AUC* luteal phase
Gastric emptying	↓ follicular phase	↓ AUC* follicular phase
Small intestine transit	↑ luteal phase	↑ AUC* luteal phase
**Cardiovascular system** Heart rate Systolic and diastolic blood pressure	↑ luteal phase ↑ luteal phase ↓ luteal phase	↑ cardiac output ↑ drug absorption
**Metabolic variation** Body weight	↑ early menstruation and following ovulation	changes in volume of distribution

*AUC: peak area (drug concentration that reaches systemic circulation at a specific time after drug administration), ↑: increase, ↓: decrease.
